# Monodisperse PEG engineering for quantifiable surface conjugation on PLGA nanoparticles

**DOI:** 10.1039/d6na00116e

**Published:** 2026-06-30

**Authors:** Ezgi Basavci, Alvja Mali, Marjan Kalati, Raphael Marques Marcilli, Mangala Srinivas

**Affiliations:** a Cell Biology and Immunology, Wageningen University and Research (WUR) Wageningen Netherlands mangala.srinivas@wur.nl; b Polypure AS Oslo Norway

## Abstract

Surface PEGylation is a well-established strategy to enhance the stability, functionality, and bioavailability of nanoparticles (NPs) in biomedical applications. However, the widespread use of polydisperse PEG derivatives limits the ability to precisely quantify surface PEGylation efficiency (mol%) and evaluate its effect on NP properties. In this study, we present a systematic approach to engineer PLGA NPs using structurally defined monodisperse PEG-diamine derivatives of varying chain lengths (PEG6, PEG26, PEG45), synthesized and purified by chromatographic methods and covalently attached *via* EDC/NHS-mediated amide bond formation under mild conditions. PEGylation was quantified by ^1^H NMR through integrating the PEG methylene and PLGA lactide signals, taking advantage of the defined chain length of the monodisperse PEGs to calculate the molar degree of PEGylation. PEGylation was strongly influenced by surface purification protocols, particularly the removal of residual surfactant (PVA). Thermogravimetric analysis (TGA) and derivative thermogravimetric analysis (DTGA) demonstrated that PEGs with higher molar mass conferred greater thermal stability to both free PEGs and PEGylated NPs. PEGylated NPs exhibited improved PFCE encapsulation (up to 18.2%) under the tested lyophilization and washing conditions. Incubation in an albumin-containing medium showed that PEGylated NPs maintained a narrow size distribution and low PDI over 20 hours, suggesting that extended PEG chains provided more effective surface shielding in a protein-rich environment. Long-term stability studies over 60 days revealed condition-dependent improvements in colloidal stability under physiologically relevant conditions. In PBMCs, all formulations maintained over 80% cell viability after 24 hours, while RAW macrophages displayed moderate formulation-dependent responses after 4 hours, with PEG45-PLGA NPs showing slightly higher viability than the other NP formulations. Overall, this work highlights that monodisperse PEG engineering enables reproducible and quantitative surface modification of PLGA NPs. This approach offers a robust platform for the rational design of advanced polymeric nanocarriers, exemplified here for ^19^F MRI applications.

## Abstract 2

Monodisperse PEG engineering enabled precise quantification of PLGA nanoparticle surface conjugation and improved stability, supporting reproducible design of PFCE-loaded nanocarriers for imaging.

## Graphical abstract

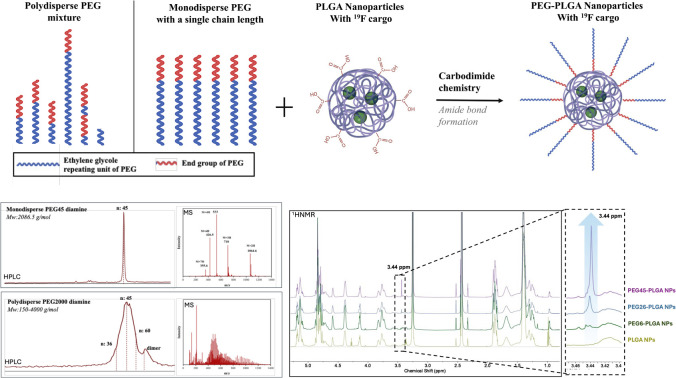

## 1. Introduction

Polyethylene glycol (PEG), also known as polyethylene oxide (PEO) or macrogol,^1^ is a polyether composed of a backbone of repeating ethylene glycol units.^1^ Its chemical stability and structural tunability (molecular weight, topology, and terminal functionality) enable diverse PEG derivatives and have made PEG important across industries, including biopharmaceuticals, cosmetics, and food technology.^2^ In biomedical settings, PEG's water solubility, hydrophilicity, and biocompatibility underpin “PEGylation” as a strategy to improve biomolecular performance.^2,3^ Davis and Abuchowski first introduced the concept of “PEGylation” in 1977, defining it as the covalent or noncovalent attachment of PEG to therapeutics or macromolecules, including oligonucleotides, peptides, proteins, and antibodies.^4,5^ PEGylation is commonly associated with “stealth” behaviour, reducing opsonization and recognition by the mononuclear phagocyte system, thereby prolonging circulation.^6^ By increasing hydrodynamic volume and solubility while reducing enzymatic degradation and immunogenicity, PEGylation can extend systemic half-life.^7^ Clinically, the first FDA-approved PEGylated product, Adagen® (the first PEGylated protein) by Enzon Pharmaceuticals, entered the market in 1990, and by 2023 more than 20 PEG-functionalized drugs had been approved, corresponding to a market exceeding 10 billion USD.^7^ Despite this success, most PEGylated drugs still rely on polydisperse PEG, primarily due to historical regulatory acceptance, large-scale availability, and established manufacturing protocols. Only a limited number employ monodisperse PEG to ensure defined molecular architecture and reproducible pharmacokinetics, including Movantik®, Zylonta®, and Trodelvy®.^7^ In parallel, reports that certain PEG-based excipients (*e.g.*, Poloxamer 188 (F68)) can induce anti-PEG IgG and IgM antibodies and trigger the accelerated blood clearance (ABC) effect have further motivated the development of well-defined monodisperse PEG architectures.^8^

Conventional polydisperse PEG is manufactured primarily through anionic ring-opening polymerization (ROP) of ethylene oxide, generating inherently heterogeneous mixtures with distributions of molecular weights and potentially different PEG species.^9^ The use of metal alkoxide initiators can contribute to uncontrolled chain termination and broad molecular weight distributions.^10^ This structural heterogeneity results in batch-to-batch variability, complicating reproducibility, purification, and precise characterization, especially in sensitive biomedical and pharmaceutical applications.^11,12^ Monodisperse PEG, also first referred to as discrete PEG (dPEG®) by Quanta Biodesign Ltd, addresses these limitations through stepwise synthetic strategies (including iterative deprotection/coupling and macrocyclic sulphate intermediates) that enable precise control over chain length.^13–15^ Chromatographic fractionation can further isolate uniform PEG oligomers with narrow dispersity, which allows batch-to-batch consistency.^16^ Such uniformity is associated with improved pharmacokinetics, biodistribution, and therapeutic performance in various NPs systems.^17–19^ These advantages are expected to translate to PLGA NPs, given the similar mechanisms of PEG-mediated surface shielding and reduced opsonization,^20,21^ and to support more predictable functionalization and biological interactions than polydisperse PEG derivatives.^17–19^

Nanoparticles (NPs) provide a versatile platform for integrating therapeutic and diagnostic functions, and PEGylation is widely used to improve colloidal stability, reduce nonspecific protein corona formation, and extend circulation time. Poly(lactic-*co*-glycolic acid) (PLGA) NPs are particularly attractive due to their biocompatibility, controlled biodegradability, and FDA-approved status.^22,23^ However, their native hydrophobic surface may lead to rapid opsonization and clearance from systemic circulation,^24^ necessitating surface engineering to improve *in vivo* performance.^24–26^ While PEGylation is a standard approach for enhancing NP stability and reducing immunogenicity,^24,27,28^ reproducible functionalization remains challenging because polydisperse PEG introduces chemical heterogeneity.^9^ This variability complicates the accurate quantification of PEGylation efficiency (% PEGylation), and chemically grounded quantification approaches remain relatively scarce in the literature.^9,12,29^ The impact of the polydispersity of PEG in the biological media, such as serum stability, is likewise insufficiently investigated. A promising route to address both challenges is the use of monodisperse PEG derivatives with engineered terminal groups.^30^ Their defined repeat-unit structure enables reliable quantification by proton nuclear magnetic resonance (^1^H NMR),^22^ and supports reproducible covalent attachment to NP surfaces. However, most PEGylated PLGA nanoparticle systems still rely on polydisperse PEG derivatives, which have broad and poorly defined chain-length distributions and higher-order byproducts, complicating quantitative analysis of surface PEG coverage and structure–function relationships. In particular, ^1^H NMR-based quantification of PEGylation is challenging when PEG is polydisperse, because the PEG signals cannot be straightforwardly related to a defined number of repeat units on the nanoparticle. Thus, there is a need for PEGylation strategies that couple monodisperse PEG architectures with analytical workflows capable of reporting PEGylation in molar terms on the nanoparticle surface.

For polymeric NPs such as PLGA NPs, carbodiimide crosslinking in aqueous environments (EDC/NHS coupling) is widely used for post-functionalization: PEG-diamine derivatives can react with surface carboxyl groups to form amide bonds.^31^ However, coupling efficiency is often limited by competing hydrolysis and is highly sensitive to reaction conditions such as pH/buffer composition, solvent environment, and activation time,^32^ as well as steric accessibility and heterogeneity of reactive sites on polymeric NP surfaces. To evaluate these limitations, robust analytical workflows are required to confirm conjugation and quantify PEGylation on NP surfaces. Spectroscopic methods such as HPLC-MS and NMR enable assessment of bond formation and coupling efficiency, while TGA provides complementary information in polymeric NP systems by tracking thermally induced mass loss to infer overall PEG content, incorporation, composition, and degradation behaviour of PEGylated and non-PEGylated PLGA NPs.^30,33^

This study focuses on optimizing PEGylated PLGA NPs (PEG-PLGA NPs) loaded with a ^19^F cargo for imaging applications, addressing key challenges in NP surface functionalization and its quantitative characterization by using well-defined monodisperse PEG derivatives. Perfluorocarbon (PFC)-loaded PLGA NPs have been extensively explored for cellular imaging by ^19^F magnetic resonance imaging (MRI), enabling quantitative signals without biological background.^34–36^ PFCs are chemically inert, fully fluorinated compounds with minimal intermolecular interactions, resulting in amphiphobic behaviour (hydrophobic and lipophobic) that supports biological inertness and strong ^19^F MRI contrast.^36,37^ Among them, perfluoro-15-crown-5-ether (PFCE) is widely used due to its symmetrical molecular structure, which results in a single, sharp ^19^F resonance, enabling highly sensitive and quantitative imaging.^36,38–40^ Although, PFCE is poorly soluble in both aqueous and lipid media, complicating formulation and encapsulation,^41^ PLGA NPs provide a compatible hydrophobic polyester matrix that supports PFCE entrapment and retention without compromising chemical stability or ^19^F signal integrity. Here, PFCE primarily serves as a convenient reporter for straightforward ^19^F NMR quantification, including in cells, while the central focus remains PLGA NP surface PEGylation.

In this study, we therefore use monodisperse PEG-diamine derivatives of defined chain lengths (PEG6, PEG26, PEG45) as quantitative internal standards for PLGA NP PEGylation and systematically examine how PEG chain length and residual PVA jointly determine the attainable surface PEG density, PFCE encapsulation, colloidal stability, and short-term *in vitro* compatibility. Moreover, PEG45-diamine serves as a monodisperse analogue of PEG_2000Da_ and is directly compared with commercial polydisperse PEG_2000Da_-diamine to assess how monodisperse *versus* polydisperse PEG architecture influences PEGylation quantification and nanoparticle performance under matched conditions. This work establishes a framework for scalable and stable nanocarriers that integrate precise PEG design with comprehensive physicochemical characterization and preliminary *in vitro* compatibility assessment.

## 2. Materials and methods

### 2.1 Materials

Triethylamine (TEA, Sigma-Aldrich), methanesulfonyl chloride (Sigma-Aldrich, 99.7%), ammonia solution (25%, Merck), acetic anhydride (Merck, 99%), Resomer® RG 502H poly(d,l-lactide-*co*-glycolide) (PLGA, Sigma-Aldrich), perfluoro-15-crown-5-ether (PFCE, Exfluor, 99%), poly(vinyl alcohol) (PVA, Sigma-Aldrich, 99%), *N*-(3-dimethylaminopropyl)-*N*′-ethylcarbodiimide hydrochloride (EDC, Thermo Scientific), and *N*-hydroxysuccinimide (NHS, Merck, 98%) were used as received. Albumin from human serum (HSA) lyophilized powder (Sigma-Aldrich, 97%). Buffer components: morpholino ethane sulfonic acid (MES) buffered saline packs (Thermo Scientific) and 1× PBS (1 mM KH_2_PO_4_, 155 mM NaCl, 3 mM Na_2_HPO_4_·7H_2_O, pH 7.4). Dialysis was performed using Spectra/Por 7 dialysis membranes (MWCO 6.5 kDa, Biotech CE tubing, pre-wet in 0.05% sodium azide). For analytical measurements, dimethyl sulfoxide-*d*_6_ (DMSO-*d*_6_, 99.8%, with TMS, MagniSolv™), deuterium oxide (D_2_O, 99.9% D, Sigma-Aldrich), and trifluoroacetic acid (TFA, 1 vol%, Sigma-Aldrich) were used. Single-length poly(ethylene glycol)-diol derivatives were obtained from Polypure AS: PEG8-diol, PEG28-diol, and PEG47-diol. Other solvents and reagents were of analytical grade and obtained from Sigma-Aldrich (Germany).

### 2.2 Methods

#### 2.2.1 Synthesis and purification of monodisperse PEG derivatives

**Fig. 1:**
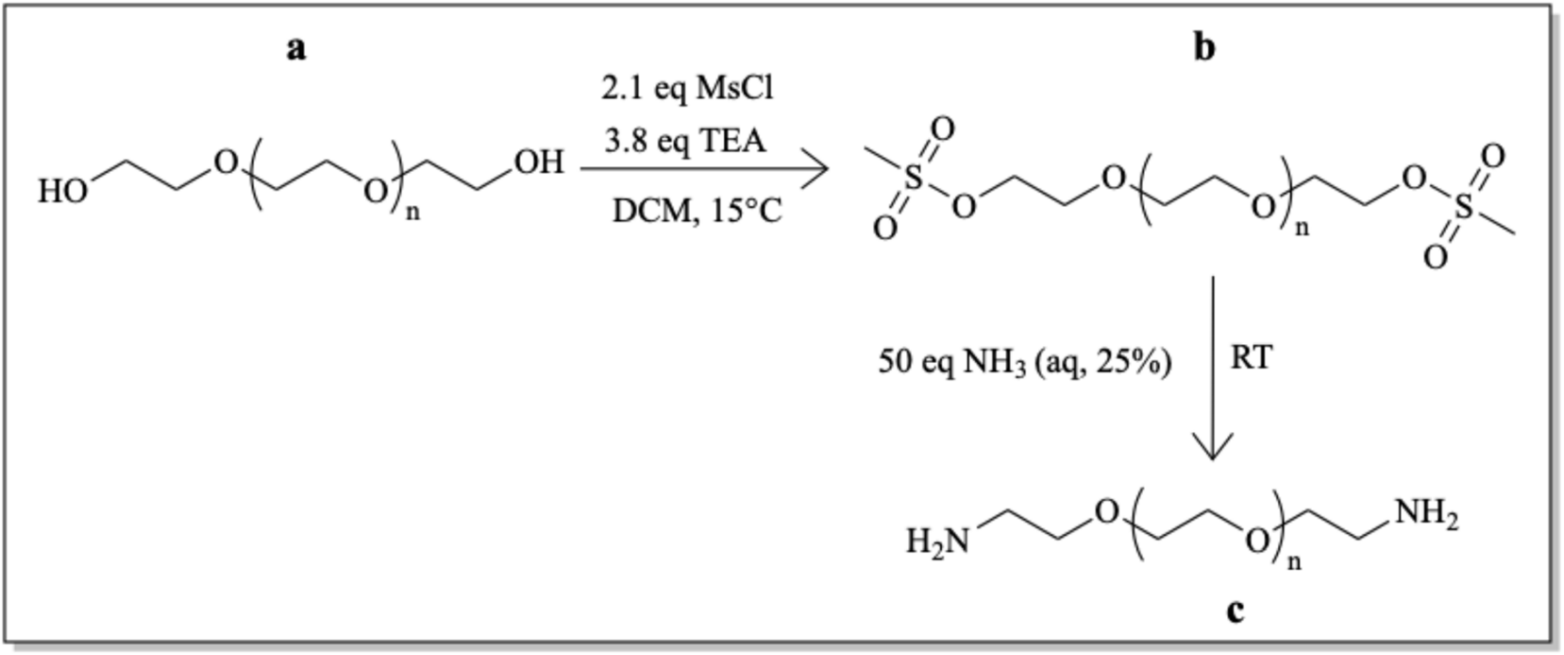
Synthesis route of monodisperse PEG‑diamines with varying lengths (*n* = 8, 28, and 47): (a) PEG‑diol mesylation, (b) conversion to PEG‑dimesylate and PEG‑diamine, (c) final monodisperse PEG‑diamine products.

The three-step synthesis of PEG-diamine derivatives was carried out under similar reaction conditions, with modifications based on the molecular weight of each PEG chain (PEG6-diamine, *M*_w_ = 368.47 g mol^−1^, PDI: 1.139; PEG26-diamine, *M*_w_ = 1249.53 g mol^−1^, PDI: 1.045; PEG45-diamine, *M*_w_ = 2086.54 g mol^−1^, PDI: 1.057) (Fig. 1c). The reaction conditions, particularly the solvent and reagent concentrations, were adjusted according to the molar mass of each PEG precursor. After mesylating and nucleophilic substitution with ammonia, two terminal ethylene oxide units were lost due to the replacement of hydroxyl groups with primary amines. Consequently, PEG8-diol, PEG28-diol, and PEG47-diol yield PEG6-diamine, PEG26-diamine, and PEG45-diamine, respectively. This naming reflects the remaining number of ethylene oxide repeat units in the PEG backbone.

##### 2.2.1.1 Synthesis of PEG-dimesylate

PEG28-diol (Fig. 1a) (400 g, 0.17 mol) was dissolved in dichloromethane (DCM, 2000 mL). Mesyl chloride (41 g, 0.53 mol) in DCM (2000 mL) was added to the polymer solution. Triethylamine (86 mL, 0.65 mol) in DCM (120 mL) was added dropwise to the reaction mixture over 30 minutes, while the reaction was cooled to precisely 15 °C. Once the reaction was complete, it was washed with 0.1 M NaHCO_3_ (1200 mL) and water (2 × 1200 mL). The organic phase was evaporated, and the resulting crude product, PEG26-dimesylate (450 g), was collected as a white solid. The same procedure was applied to PEG8-diol (370.4 g mol^−1^) and PEG47-diol (1251.5 g mol^−1^) with appropriate adjustments in reagent concentrations based on the molecular weights of the PEG chains.

##### 2.2.1.2 Synthesis of PEG-diamine

PEG26-dimesylate (Fig. 1b) (540 g, 0.384 mol) was mixed with aqueous ammonia (25% w/w NH_3_) (1279 mL, 17 mol). The reaction mixture was stirred for two days at room temperature (rt). The product mixture was extracted with DCM (3 × 200 mL). The three DCM fractions were evaporated and then subjected to a patented preparative chromatographic purification system (sample displacement chromatography) at Polypure AS, Norway. Finally, pure diamine fractions were collected and extracted with DCM from an aqueous basic solution. The same procedure was applied to PEG8-dimesylate (526.6 g mol^−1^) and PEG47-dimesylate (2244.7 g mol^−1^), with appropriate adjustments in reagent concentrations based on the molecular weights of the PEG chains.

#### 2.2.2 Synthesis of PLGA NPs and their post-PEGylation

##### 2.2.2.1 Preparation of ^19^F-based contrast agent entrapped poly(lactic-*co*-glycolic acid) NPs (PLGA NP)

Particles were prepared and characterized as in ref. 42. PLGA (100 mg) was dissolved in DCM (3 mL), followed by the addition of PFCE (900 µL), resulting in a double-phase liquid. This mixture was rapidly added with a glass pipette to the solution of PVA (25.5 g, 1.96 wt% solution in ultra-pure H_2_O) in a round-bottom flask. During the addition, the PFCE and PLGA mixture in DCM was pipetted up and down to ensure both phases were added simultaneously. The mixture was sonicated in an ice-water bath for 3 min at an amplitude of 40% (microtip Branson digital sonifier s250). After sonication, DCM was evaporated at 4 °C overnight under stirring to solidify the particles. The particles were washed by centrifugation at 16 087 G for 35 min in 50 mL Falcon centrifugation tubes and resuspended in 25 mL of water. Variations in the PVA washing steps resulted in different washed PLGA NPs, including those washed once, twice, or thrice. After the desired washing steps, the NPs were either resuspended in 4 mL of water, frozen using liquid nitrogen, and subsequently freeze-dried (referred to as PLGA NP-solid), or resuspended in 8.5 mL of MES buffer and stored at 4 °C (referred to as PLGA NP-in-solution). The freeze-dried PLGA NPs were obtained as a white powder, with a typical final yield of 85 mg.

##### 2.2.2.2 Post-PEGylation on the surface of nanoparticles (PEG-PLGA NP)

PLGA NP-solid (30 mg, 0.0022 mmol) was either resuspended in MES buffer (3 mL, 0.1 M, pH 4.5–5) or PLGA NP-in solution (3 mL in MES buffer) was used as the pre-dispersed solution. EDC (3.3 mg, 0.017 mmol) and NHS (2.5 mg, 0.0214 mmol) were added to the solution. The reaction mixture was stirred for 45 min at rt. PEG-diamine (0.054 mmol) was dissolved in PBS (1 mL, pH ∼7.4) and added to the reaction mixture. Different PEG chain lengths were also used, with concentrations adjusted according to their molecular weights. The resulting mixture was stirred overnight and then purified by dialysis using a dialysis bag with a molar weight cut-off of 6.5 kDa, in PBS solution (24 hours), followed by water (at least 48 hours). The dialysis solution was changed thrice daily to remove excess PEG, EDC, NHS, and urea from the resulting solution. The washed NPs were frozen in liquid nitrogen and freeze-dried for 72 hours.

Throughout this work, we use PEGylation to denote the attachment of PEG-diamine to PLGA NP surface carboxyl groups under EDC/NHS coupling conditions; PEGylation efficiency to denote the quantitative yield of this reaction as determined by ^1^H NMR (eqn (1)); and surface conjugation as a synonym for this surface modification.

#### 2.2.3 Analytical techniques and instruments

##### 2.2.3.1 High-performance liquid chromatography-mass spectrometry (HPLC-MS)

HPLC-MS confirmed the oligomeric purity and molecular weights of the synthesized PEG derivatives by matching the observed *m*/*z* peaks with calculated values. The analysis was performed on an Agilent 1260 Infinity LS system coupled to an Agilent 6110 MS Single Quadrupole equipped with electrospray ionization (ESI). An ACE 3C18 column (30 mm × 2.1 mm, 3 µm particle size, VWR) was used for separation at a column temperature of 22 °C. The mobile phase consisted of water (0.1% TFA) as solvent A and acetonitrile as solvent B, with a gradient elution profile as follows: 5% B at 0 min, increasing to 45% B over 10 min. The flow rate was set to 0.2 mL min^−1^, and the injection volume was 1 µL.

In positive ion mode, mass spectrometry data were acquired using electrospray ionization (ESI). The mass spectrometer parameters were set as follows: the capillary voltage was 3 kV, high-purity nitrogen (99.99%) was used as both the nebulizing and drying gas; the nebulizer pressure was 35 psi, the drying gas flow rate was 12 L min^−1^, and the drying gas temperature was 349 °C. Data were acquired in full-scan mode with a mass range of 100–2000 *m*/*z*. The resulting spectra were analysed using Agilent ChemStation software to determine the molecular weight and the architecture of the PEG derivatives.

##### 2.2.3.2 Gel permeation chromatography (GPC)

Relative number average molar mass (*M*_n_), weight average molar mass (*M*_w_), and dispersity (*Đ*_M_ = *M*_w_/*M*_n_) were determined by gel GPC performed on Agilent RI, LALS/RALS, and DP/IP multi detectors (670 nm laser as light source). The mobile phase used was PSS polystyrene in THF. All analyses were performed at 1.0 mL min^−1^. Samples were dissolved in THF at 5 mg mL^−1^. Before injection, all samples were filtered through 0.45 µm PVDF filters (Whatman). A calibration curve was constructed using polystyrene standards (Agilent) with molar mass ranging from 1820 to 2005000 g mol^−1^.

##### 2.2.3.3 Proton nuclear magnetic resonance (^1^H NMR)

^1^H NMR spectroscopy was used to verify the chemical structure of the polymers and to assess the extent of PEG incorporation (PEGylation efficiency, eqn (1)) through quantitative analysis of characteristic proton signals. ^1^H NMR spectra were recorded using a 700 MHz Bruker Biospin NMR spectrometer, equipped with a Broadband Inverse (BBI) probe, at a temperature of 298 K. Approximately 8–10 mg of a polymer was dissolved in 600 µL of deuterated dimethyl sulfoxide (DMSO-*d*_6_) and transferred to 5 mm NMR tubes. Data acquisition was performed with 30 scans, using a relaxation delay of 45 seconds to ensure complete relaxation for accurate quantification. Data analysis was carried out using Mestrenova 14.3.0 software.

##### 2.2.3.4 Fluorine-19 nuclear magnetic resonance (^19^F NMR)

Quantitative analysis of the fluorine content in PFCE-encapsulated PLGA NPs was performed by ^19^F NMR spectroscopy. A defined amount of the NPs was resuspended in 500 µL of D_2_O and 100 µL of 1 vol% TFA, used as an internal reference. The prepared solution was transferred into 5 mm NMR tubes, and the analysis was performed on a Bruker Avance III 400 MHz NMR spectrometer equipped with a BBFO + probe. Quantitative ^19^F NMR data were acquired with an interscan relaxation delay of 25 seconds, and 8 scans were performed to improve signal averaging and ensure accurate quantification. Data analysis was performed using Mestrenova 14.3.0 software, enabling the precise integration of the fluorine signals and calculation of the fluorine content in the NPs.

##### 2.2.3.5 Dynamic light scattering-zeta potential (DLS-zeta)

The particle size distribution, polydispersity index (PDI), and zeta potential of the PEGylated PLGA NPs were determined using Dynamic Light Scattering (DLS) and zeta potential analysis. NPs were first dispersed in Milli-Q (MQ) water at a concentration of 10 mg mL^−1^. This stock solution was diluted 100-fold to a final concentration of 0.01 mg mL^−1^, with a total volume of 1 mL. DLS and zeta potential measurements were performed on a Malvern Zetasizer Nano ZS (Malvern Panalytical Ltd, UK) with disposable cuvettes. The particle size distribution and PDI were measured at a scattering angle of 173° at 25 °C, while zeta potential measurements were conducted at 21 °C. Data acquisition for each sample involved four independent measurements, each consisting of six runs of 10 second duration.

##### 2.2.3.6 Thermogravimetric analysis (TGA)

Thermogravimetric analysis (TGA) was used to assess the thermal stability and decomposition profiles of the polymers and NPs. Approximately 5–8 mg of dried samples were placed in a platinum pan and analysed using a TGA Q500 (TA Instruments) under a nitrogen atmosphere with a flow rate of 50 mL min^−1^. The temperature was ramped from 35 °C to 800 °C at a heating rate of 10 °C min^−1^. The weight loss was recorded as a function of temperature, and a derivative thermogravimetric (DTGA) curve was used to identify key thermal events, such as the degradation of PLGA and PEG segments.

#### 2.2.4 *In vitro* serum stability test with albumin

The interaction between albumin (HSA) and PLGA NPs, PEG26-PLGA NPs, and PEG45-PLGA NPs was evaluated by monitoring changes in Z-average size (nm) and polydispersity index (PDI) over a 24 hour period in an albumin solution with a concentration of 35 mg mL to mimic physiological serum protein levels (∼55% w/w). For each formulation, NPs (3 mg) were incubated in 1 mL of the albumin solution at 37 °C in 2 mL Eppendorf® tubes using a Thermomixer (350 rpm). Samples were collected at 0 min, 5 min, 2 h, 6 h, and 20 h, and analysed with a Malvern Zetasizer Nano.

#### 2.2.5 Long-term stability testing

Nanoparticles, PEGylated and non-PEGylated, were suspended in two environments: deionized water and Dulbecco's Modified Eagle Medium (DMEM) at a concentration of 5 mg mL^−1^. The NP suspensions were transferred into a 24-well plate, with each well containing 500 µL of the suspension. To ensure proper dispersion of the NPs, the samples were subjected to sonication in an ultrasonicator for 5 minutes. Following sonication, the samples were incubated under three temperature conditions: one set of wells was maintained at 37 °C, mimicking physiological conditions, another at 25 °C, as room temperature, and a third set was stored at 4 °C to evaluate the effect of different storage conditions on stability. Samples were collected at 0, 3, 7, 10, 17, 30, and 60 days for all conditions. Before each DLS measurement, the samples were vortexed and briefly sonicated again to ensure homogeneity. DLS measurements were conducted in triplicate for each sample to evaluate changes in NP size and PDI over time.

#### 2.2.6 Cytotoxicity in cells

Cytotoxicity in cells was evaluated using RAW 264.7 murine macrophages and human peripheral blood mononuclear cells (PBMCs). RAW 264.7 cells were obtained from ATCC and cultured in DMEM supplemented with 10% foetal bovine serum (FBS) and 1% penicillin/streptomycin at 37 °C in a humidified atmosphere containing 5% CO_2_. Buffy coats from healthy donors were obtained from Sanquin Blood Bank (Nijmegen, The Netherlands) after written informed consent and in accordance with its ethical guidelines. All experiments were carried out in accordance with the ethical guidelines of Sanquin Blood Bank and Wageningen University & Research, as well as with national and institutional regulations. Wageningen University approved the experiment. PBMCs were purified from the buffy coats by Ficoll density gradient centrifugation using Leucosep tubes, following a previously described protocol^43^ and cultured in RPMI 1640 supplemented with 10% foetal bovine serum (FBS) and 1% penicillin/streptomycin at 37 °C in a humidified atmosphere containing 5% CO_2_.

For cytotoxicity studies, cells were incubated with the indicated nanoparticles at 5 mg mL^−1^ for the exposure times specified in the corresponding figure legends. Cell viability was then determined by flow cytometry using Zombie Aqua staining.

#### 2.2.7 Statistical analysis

Statistical analyses were performed using GraphPad Prism version 10.3.0 for macOS (GraphPad Software, Boston, Massachusetts, USA). One-way ANOVA followed by Tukey's multiple comparisons test was applied to compare differences among groups. Data are presented as mean ± standard deviation (SD) from at least three independent samples (*n* ≥ 3). Error bars in graphs represent SD, and statistical significance was defined as *p* < 0.05.

## 3 Results and discussion

Commercial PEGs are largely used in pharmaceuticals and clinical products, including anticancer drugs and mRNA vaccines.^44,45^ However, PEG polydispersity introduces practical limitations for downstream bioconjugation: heterogeneous chain populations complicate analytical characterization, reduce batch-to-batch consistency, and hinder precise control over chemical reactivity. Hermanson *et al.* emphasized that a polydisperse PEG mixture impairs reproducibility and may reduce cellular uptake in some contexts, thereby lowering bioconjugation efficiency.^46^ In addition to these technical constraints, PEG is no longer viewed as universally non-immunogenic. Reports of anti-PEG antibodies have increased markedly, from 0.2% in 1984 to ∼72% in 2016, and around 7% of individuals exhibit antibody levels that might be high enough to potentially trigger allergic reactions.^47,48^ More recent comprehensive analyses confirm sustained high prevalence across diverse populations.^49^ Rare cases of anaphylaxis associated with PEG2000-containing lipid excipients used in mRNA vaccines have further highlighted concerns about PEG-related hypersensitivity.^19,50^ Together, these considerations motivate the development of monodisperse, single-length PEGs, which provide a defined molecular weight and reduced low-molecular-weight species and reactive byproducts, thereby improving quantifiability and enabling more reproducible and controlled surface functionalization.^51^ Replacing polydisperse PEG mixtures with monodisperse, single-length PEG reduces heterogeneity, improves reproducibility, and can enhance therapeutic performance by lowering variability in biodistribution and immune responses.^17,52^

In addition to PEG dispersity, residual PVA is a critical parameter in this system because it can substantially influence PLGA NP surface properties. During emulsification, PVA acts as a stabilizer, but it can remain adsorbed on the NP surface or entrapped within the polymer matrix after preparation, and incomplete removal can interfere with surface PEGylation efficiency. Therefore, in this work we systematically varied the PVA washing protocols and evaluated how both PEG chain length and residual PVA removal together shape PEGylation efficiency and NP stability under the tested conditions.

### 3.1 Synthesis and characterization of monodisperse PEG derivatives

Despite numerous advances in the synthesis of monodisperse PEGs, producing high molecular weight monodisperse PEGs at a preparative scale (repeating units (*n*) ≥ 32, corresponding to ≥∼1500 Da) remains a significant challenge.^1^ Prior studies have reported various strategies, such as A + B + A type etherification reactions, macrocyclic sulphate intermediates, stepwise solid-phase growth methods, and Williamson ether synthesis, to access PEG chains up to 64 repeating units.^53–56^ However, these methods often require multiple protection/deprotection and purification steps and can suffer from low overall yields, especially for PEGs above 1000 Da. Another synthetic approach uses strong bases like sodium hydride (*e.g.*, NaH), which further increases the risk of side reactions, limiting the efficiency and scalability.^13^ Here we use a process developed at Polypure AS in which longer PEG-diol chains (containing *n* ≥ 47 monomeric units per chain, corresponding to ≥∼2000 Da) can be purified at a preparative scale (up to kilogram scale) with very high oligomeric purity. Fig. 1 outlines the synthesis route for PEG-diamine derivatives from PEG-diol and the chromatographic purification workflow used to obtain defined PEG chain lengths and end groups functionality for subsequent NP conjugation. Using this approach, monodisperse PEG-diamines of varying chain lengths were successfully engineered (PEG6-diamine; *M*_w_: 368.47 g mol^−1^, PEG26-diamine; *M*_w_: 1249.53 g mol^−1^, and PEG45-diamine; *M*_w_: 2086.54 g mol^−1^), and their HPLC-MS and ^1^H NMR analyses confirmed high purity and well-defined structures, ensuring the reproducibility and consistency needed for subsequent conjugation to PLGA NPs.

**Fig. 2:**
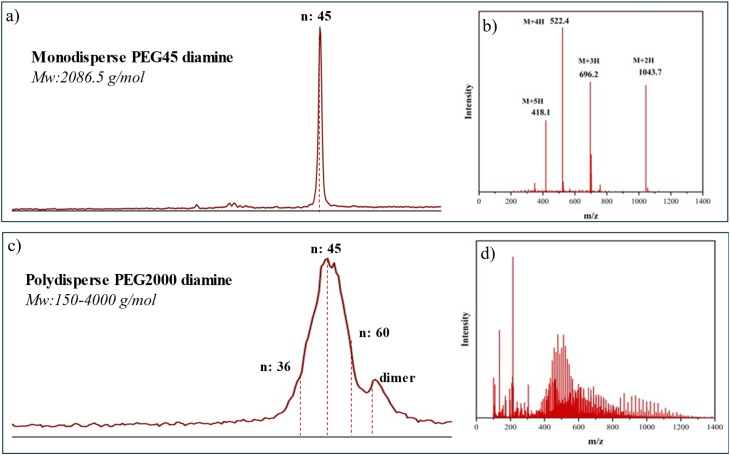
HPLC chromatograms of (a) monodisperse PEG45 diamine and (c) polydisperse PEG_2000Da_ diamine. The mass spectrum belonging to the HPLC-ESI MS test is (b) monodisperse PEG45 diamine and (d) polydisperse PEG_2000Da_ diamine.

Commercial PEG_2000Da_-diamine is widely used as benchmarks in PEGylation studies. The present study presents a comparison of PEG_2000Da_ (standard) with the synthesized monodisperse PEG45 to highlight differences in analytical and structural properties. HPLC-MS analysis revealed distinct differences in molecular weight distribution between the synthesized PEG45-diamine and commercial PEG_2000Da_-diamine samples. As shown in Fig. 2, the precisely engineered PEG45 analogue of PEG_2000Da,_ with 45 repeating ethylene glycol units and a defined molecular weight of 2086.54 g mol^−1^, exhibited a sharp, singular peak at HPLC chromatogram, confirming its monodisperse nature (Fig. 2a). In contrast, commercial PEG_2000Da_-diamine displayed a broad and heterogeneous profile, encompassing polymer chains ranging from PEG36-diamine (*M*_w_: 1500 g mol^−1^) to PEG60-diamine (*M*_w_: 4000 g mol^−1^), including higher-order dimeric species (Fig. 2c). The uniformity of PEG45-diamine makes it a particularly useful reference for accurate quantification of PEGylation yields *via*^1^H NMR, as applied here to determine PEG conjugation on PLGA NPs.

The ESI-MS spectrum of PEG45-diamine (Fig. 2b) showed distinct peaks corresponding to its multiple charge states, including [M + 2H]^2+^ at 1043.7 *m*/*z*, [M + 3H]^3+^ at 696.2 *m*/*z*, [M + 4H]^4+^ at 522.4 *m*/*z*, and [M + 5H]^5+^ at 418.1 *m*/*z*. These charge states verified the molecular weight of 2086.5 g mol^−1^ with high precision. In contrast, the commercial PEG_2000Da_-diamine (Fig. 2c) displayed a broad and unresolved chromatographic peak consistent with a heterogeneous mixture of chain lengths. This was supported by the mass spectrum (Fig. 2d), which revealed a wide range of polymer species, with charge states corresponding to PEG35 ([M + 3H]^3+^ at *m*/*z* 515.9) and PEG60 ([M + 3H]^3+^ at *m*/*z* 898) among others. Consistent with the expected ESI-MS behaviour of polydisperse polymers, overlapping ion series from multiple chain lengths produced unresolved signals. As a result, individual oligomers could not be reliably resolved, complicating accurate chain-length quantification for subsequent conjugation.

The polymer architecture and chain lengths of monodisperse PEG derivatives were determined by HPLC-MS (Fig. S1a–S3a) and ^1^H NMR spectroscopy (Fig. S1b–S3b). The integration of the peaks assigned to the terminal methylene protons adjacent to the amine groups (–C**H**_**2**_–NH_2_ at *δ* 2.4 ppm) was consistently set to four protons, serving as an internal reference. Considering the integral of characteristic methylene hydrogens, assigned to the ethylene glycol repeating units (–C**H**_**2**_C**H**_**2**_O–) at *δ* ∼3.44 ppm, we calculated the ratio (–C**H**_**2**_C**H**_**2**_O–/–C**H**_**2**_NH_2_) to determine the number of monomeric units for each chain and its molar mass (*M*_n_). Specifically, PEG6 exhibited 24 protons (Fig. S1b), PEG26 showed 104 (Fig. S2b), and PEG45 showed 180 (Fig. S3b), which matched the expected proton counts (4 × *n*) based on the number of ethylene glycol units. In contrast, the commercial polydisperse PEG_2000Da_ sample had a broader and less defined signal, with a calculated integral corresponding to 220 protons, reflecting its heterogeneous mixture of chain lengths, which may vary by up to 55 repeating ethylene glycol units (Fig. S4). The well-defined and consistent signals in the synthesized derivatives underscore their high purity and structural integrity, further supporting their suitability for precise applications.

Based on these results, although PEG45 and commercial PEG_2000Da_ have comparable average molecular weights and are subsequently conjugated to PLGA NPs under identical EDC/NHS coupling conditions and evaluated using the same characterization workflow, they are not fully equivalent. PEG45 is a structurally defined monodisperse PEG with a fixed number of 45 ethylene glycol units, whereas commercial PEG_2000Da_ is polydisperse and contains a distribution of chain lengths centred on a similar average but spanning from ∼PEG36 to ∼PEG60, as well as higher-order byproducts including dimeric species (Fig. 2c). Therefore, comparisons between PEG45-PLGA NPs and PEG_2000Da_-PLGA NPs should be interpreted as evaluating the combined effect of PEG structural definition, dispersity, and byproduct content, rather than molecular weight alone. In contrast, chain-length effects can be examined directly within the monodisperse series PEG6, PEG26, and PEG45, where dispersity and synthetic origin are held constant.

### 3.2 Post-PEGylation of PLGA NPs and quantification

**Scheme 1:**
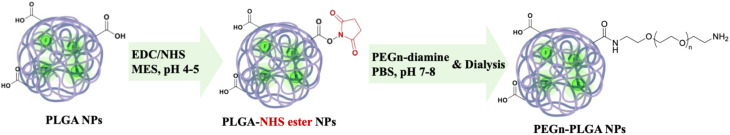
Illustration of a preparation path of PEG-PLGA NP by carbodiimide chemistry on the surface of PLGA NPs.

Firstly, PFCE-encapsulated PLGA NPs were prepared, followed by surface modification to improve colloidal stability and enable precise functionalization using synthesized single-length PEG derivatives (PEG6-diamine, PEG26-diamine, and PEG45-diamine). In addition, commercial PEG_2000Da_-diamine was included as a reference for PEG45-diamine under matched conditions. As illustrated in Scheme 1, PEGylation was performed *via* carbodiimide coupling in aqueous buffer solutions. Briefly, surface-exposed PLGA carboxyl terminal groups were activated in MES buffer (pH 4 and 5) using EDC and NHS to form an NHS-ester intermediate (PLGA-NHS ester NPs). Amine-terminated PEG derivatives in PBS (pH ∼7) were then added to promote nucleophilic substitution of the primary amine in the NHS-activated carboxylate and subsequent stable amide bond formation, yielding PEGn-PLGA NPs with amine groups. The resulting formulations were purified *via* dialysis (molecular-weight cut-off 6.5 kDa). This strategy yielded a series of PLGA-NP conjugated with PEG-diamines of different chain lengths (PEG6-PLGA NPs, PEG26-PLGA NPs, PEG45-PLGA NPs, PEG_2000Da_-PLGA NPs), providing a platform to evaluate the influence of polymer size on surface functionalization, stability, and biological performance.^57–59^

**Fig. 3:**
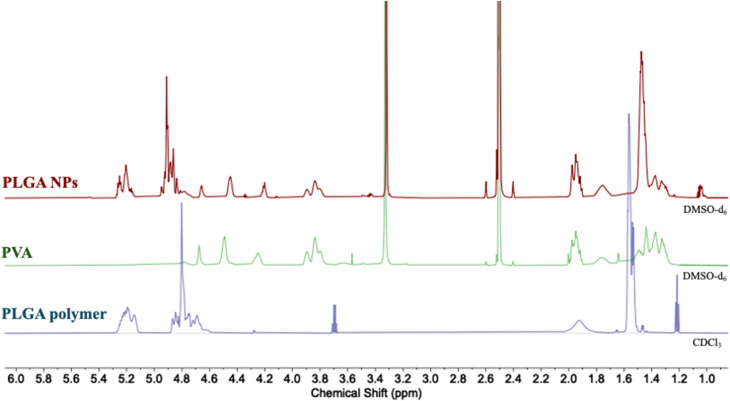
Overlay of the ^1^HNMR spectra of PLGA NP (red), PVA (green), and PLGA polymer (blue).

Accurate quantification of PEGylation required characterization of the complete NP composition, including PVA (stabilizer), PLGA (Resomer® 502H), and PFCE. Therefore, ^1^H NMR spectra of the individual components and the assembled NPs were overlaid and analysed to assess spectral contributions and potential changes associated with amide bond formation (Fig. 3). In the PLGA NPs spectrum (red, Fig. 3), characteristic peaks at *δ* 5.2 ppm and *δ* 4.8 ppm were assigned to the lactic and glycolic acid unit protons, respectively, of the PLGA backbone. Although the PVA and PLGA polymer signals overlapped in the *δ* 1 and 2 ppm region, a distinct peak at 1.5 ppm, assigned to the methyl (–C**H**_**3**_) protons of lactic acid, remained identifiable. Additionally, signals between *δ* 1.6–4.6 ppm confirmed the presence of PVA in the final composition, demonstrating that both polymers contribute to the overall spectral profile of the PLGA NPs.

Fig. S5 displays the ^1^H NMR spectrum of the commercially available PLGA polymer Resomer® RG 502H, which was used in this study to formulate the NPs. Previous ^1^H and ^13^C NMR studies,^60–62^ along with the Evonik technical datasheet, describe RG 502H as having a near-random monomer distribution, with a blockiness value (*R*_c_) of approximately 1.4. This indicates that lactic acid (L) and glycolic acid (G) units are statistically distributed throughout the polymer chain rather than forming long uninterrupted blocks. This microstructural pattern reflects the inherent kinetics of ring-opening polymerization (ROP), where the differing reactivity of L and G monomers leads to a random distribution of monomer units along the polymer chain.^22,60^

According to the manufacturer, Resomer® RG 502H is a carboxylic acid-terminated PLGA with a 50 : 50 lactide:glycolide molar ratio and a number-average molecular weight (*M*_n_) of approximately 14 000 Da (GPC; Evonik technical datasheet, Lot No: BCBZ79). Accurate quantification of PEGylation by ^1^H NMR required estimating the number of PLGA repeating units contributing to the relevant integrals. Based on the monomer masses of lactide (72.06 g mol^−1^) and glycolide (58.05 g mol^−1^) and assuming a 50 : 50 composition, the PLGA chain contains an estimated 107 to 108 repeating units of each monomer. For simplicity in signal integration and normalization across samples, the number of repeating units was approximated as 100 L and 100 G units. Monodisperse single-length PEGs were therefore synthesized and used as defined quantitative internal standards for PEG-PLGA NPs. PEGylation efficiency was determined for each NP formulation using the corresponding ^1^H NMR spectra and integration data (Fig. S6–S8), enabling calculation of the degree of PEGylation (mol% of PEG-functionalized PLGA; eqn (1)).1
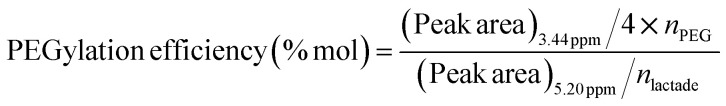


This quantitative framework allows comparison of PEGylation levels across different PEG chain lengths and formulations on a molar basis, rather than relying on indirect or qualitative measures of surface modification.

Briefly, PEGylation efficiency was calculated by normalizing the PEG methylene signal to the number of protons contributing to this resonance and to the defined PEG chain length, using the PEG methylene signal at *δ* 3.44 ppm and the lactide methine signal at *δ* 5.20–5.22 ppm as described above.

For example, in Fig. S8, the integral assigned to the PEG methylene protons was 17.2. This value was divided by 4, corresponding to the four protons per ethylene glycol repeating unit, and then divided by 45, corresponding to the number of repeating units in PEG45. The resulting value was then divided by the normalized PLGA lactide signal, calculated from the integral of the lactide methine proton, which was 100, divided by the estimated average number of lactide units in PLGA (approximated as 100, as described above). This normalization allowed the PEG signal to be expressed relative to the PLGA backbone and enabled comparison among PEG derivatives of different chain lengths.

A key strength of this study lies in the use of monodisperse PEG derivatives, for which the ethylene glycol proton integral matches the theoretical proton count for each defined chain length, enabling consistent, molar quantification of PEGylation across formulations. In contrast, conventional polydisperse PEGs such as commercial PEG_2000Da_-diamine are mixtures consisting of many different chain lengths and higher-order byproducts, which makes calculation of PEGylation efficiency by ^1^H NMR more ambiguous and less reliable, thereby contributing to significant batch-to-batch variability and weaker structure–function correlations. Thus, the monodisperse nature of PEG in this study not only facilitated precise PEGylation quantification on PFCE-loaded PLGA NPs but also demonstrated a key methodological advantage over conventional PEG systems, addressing a commonly overlooked limitation in the literature on NP functionalization.

**Fig. 4:**
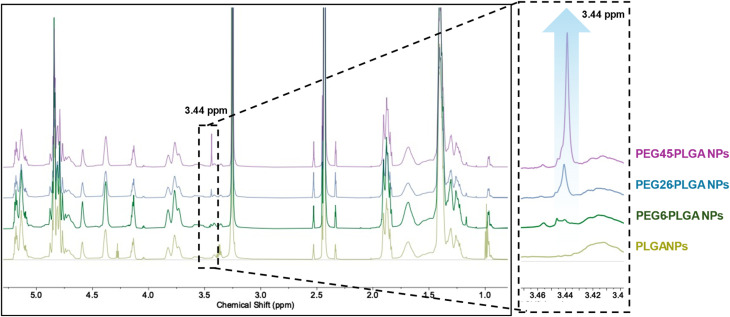
Overlay of ^1^H NMR spectra of dPEG45-PLGA NP (purple), dPEG26-PLGA NP (blue), dPEG6-PLGA NP (dark green), and PLGA NP (light green).

As shown in Fig. 4, the prominent peak at *δ* 3.4 ppm exhibited increased integration values proportional to the number of protons present in the lactide repeating units in the PLGA polymer. Among the tested PEG chain lengths, PEG45 demonstrated the highest integration (purple, Fig. 4), followed by PEG26 (blue, Fig. 4) and PEG6 (dark green, Fig. 4), clearly reflecting the ability to track chain length through ^1^H NMR analysis.

PEGylation efficiency was determined for each NP formulation individually. The mean ± SEM values were 13 ± 2.4% for PEG6-PLGA NPs, 10 ± 1.6% for PEG26-PLGA NPs, 11 ± 3.6% for PEG45-PLGA NPs, and 19 ± 3.4% for PEG_2000Da_-PLGA NPs, based on 9 to 20 independently prepared NP batches per group. This relatively narrow variation range indicates consistent conjugation efficiency across different PEG lengths. This relatively narrow range suggests reproducible coupling across compositions. The similar PEGylation efficiencies for PEG6, PEG26, and PEG45 indicate that, under our mild EDC/NHS conditions, increasing chain length does not strongly penalize conjugation efficiency, suggesting that steric hindrance is not the dominant factor determining coupling yield. By contrast, the somewhat higher apparent PEGylation of PEG_2000Da_ may partly reflect its polydispersity and broader distribution of reactive species, which can complicate direct comparison of mol% values between monodisperse and polydisperse PEGs. Slight variations in PEGylation efficiency may partly reflect the intrinsic polydispersity of PLGA, the approximation of PLGA repeat units used for ^1^H NMR normalization, and formulation-dependent differences in NP composition. The PEGylation efficiencies reported here were obtained after extensive purification/washing to reduce the contribution of residual formulation components and weakly associated species. As shown later in the manuscript, the apparent PEGylation percentage can vary depending on washing conditions, highlighting the importance of controlling this step.

Variations in PEGylation efficiency may also stem from the nature of the interaction between PEG and PLGA, which can be either covalent or noncovalent, depending on the reaction conditions. In the case of noncovalent interactions, PEG chains are only loosely associated with the NP surface and can detach during dialysis purification, particularly under physiological conditions.^63,64^ In this context, the possible contribution of residual formulation components, particularly PVA, to signal integration and surface accessibility was further evaluated through the washing experiments described in the following section. These differences could also be attributed to technical parameters, including the type of dialysis membrane (MWCO) used and the duration of dialysis in either PBS or water. In the literature, these factors have been shown to influence the yield of surface modification reactions in colloidal systems, especially under mild coupling conditions such as EDC/NHS-mediated conjugation.^65^ These results are consistent with studies on other carboxyl-functionalized systems, such as cellulose nanofibrils, where EDC/NHS-mediated PEGylation was also found to be influenced by reaction and purification conditions. In that study, even as little as 1% PEGylation significantly improved colloidal stability, particularly when longer PEG chains were used.^66^ This emphasizes the critical role of both PEG length and experimental setup in achieving effective and stable surface modification.

To complement the quantitative insights obtained from ^1^H NMR analysis, TGA was employed to assess the PEGylation of PLGA NPs qualitatively. As a well-established technique in polymer characterization, TGA offers valuable information on the thermal stability and composition of polymeric systems. Thermal analysis of polymer formulations can reveal differences in PEG chain length and content, as these factors influence the degradation profile and overall thermal stability of the material. In particular, the first derivative of the TGA curve (DTGA) highlights the thermal events associated with the different polymers over specific temperature intervals, offering qualitative confirmation of PEG conjugation.

**Fig. 5:**
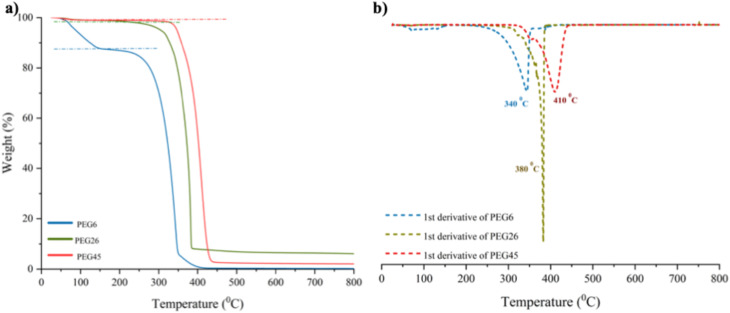
(a) TGA thermogram in inert atmosphere and (b) DTGA graphs of PEG derivatives.

In Fig. 5, the TGA curves of PEG derivatives displayed major degradation events in the range 340 °C to 410 °C, associated with thermal decomposition of PEG, with the event shifting to higher temperatures as molar mass increased, indicating chain-length-dependent thermal stability. PEG6 exhibited an onset degradation temperature of approximately 220 °C, indicating the initiation of thermal decomposition at a relatively lower temperature. In contrast, PEG26 and PEG45 showed higher onset degradation temperatures of approximately 280 °C and 300 °C, respectively, reflecting enhanced thermal stability with increasing PEG chain length (Fig. 5a). The early weight-loss step observed for PEG6 around 100–150 °C can be attributed to its liquid state at room temperature and its hygroscopic nature, which may facilitate evaporation of residual moisture. This initial mass loss could also reflect residual solvents or absorbed water within the PEG6 sample, which can evaporate or decompose at lower temperatures. In contrast, PEG26 and PEG45 did not exhibit this early weight-loss step, as both are solid at room temperature, which is consistent with higher thermal stability and higher onset degradation temperatures. This highlights the significant influence of the physical state of PEG, as well as potential solvent or water content, on the observed thermal behaviour. DTGA analysis provided further insight into the thermal degradation behaviour. PEG6-diamine exhibited a DTGA maximum at approximately 340 °C, while PEG26-diamine and PEG45-diamine displayed maxima at higher temperatures, around 380 °C and 410 °C, respectively (Fig. 5b). These DTGA maxima indicate thermal decomposition of the PEG segments, confirming that longer PEG chains degrade at higher temperatures.

**Fig. 6:**
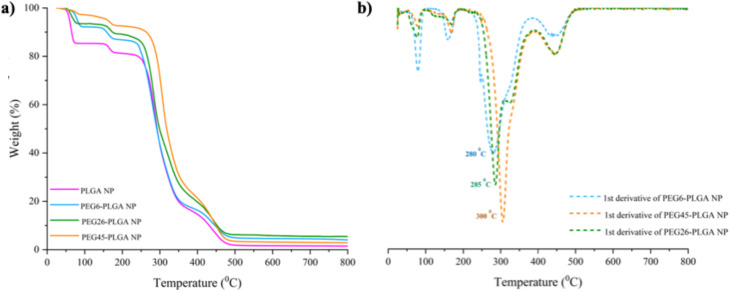
Thermogravimetric curves acquired under inert atmosphere: (a) TGA and (b) DTGA curves of PEG6-PLGA NP, PEG26-PLGA NP, PEG45-PLGA NP.

PEG-PLGA NPs revealed a clear shift in thermal degradation behaviour depending on PEG chain length (Fig. 6a). In the first derivatives (Fig. 6b), the major decomposition event shifted to higher temperatures with increasing PEG length, with onset temperatures from 280 °C for PEG6-PLGA NPs, to 285 °C for PEG26-PLGA NPs, and to 300 °C for PEG45-PLGA NPs. This progressive shift is consistent with the thermal degradation ranges for pure PEGs (Fig. 5), which typically exhibit a single-step (monomodal) degradation profile with a peak temperature in the range of 340–410 °C, depending on the molecular weight. This trend suggests that increasing PEG chain length may enhance the thermal stability of PEGylated NPs, likely due to stronger intermolecular interactions, changes in polymer packing density, and differences in thermal transitions (*e.g.*, glass-transition temperature, *T*_g_). Previous works have reported a similar trend, showing that PEGylation affects the crystallinity and thermal stability of polymeric systems, where longer PEG chains result in higher melting and degradation temperatures.^67,68^ Complementarily, increasing PEG content or using lower molecular weight PEGs has been reported to reduce the onset temperature of thermal degradation in polymer blends, as observed in poly(lactic acid)/poly(ethylene glycol) and poly(ε-caprolactone)/poly(ethylene glycol) systems.^61,62^ These findings indicate that PEG structure can significantly influence thermal stability and may also affect the PEG retention on surface-functionalized NPs; for example, higher molar mass PEG may be retained more effectively due to an increased probability of intra- and inter-chain physical entanglements.

Additionally, as shown in Fig. S9, DTGA analysis revealed that the maximum degradation of the PLGA polymer occurred at approximately 310 °C, while PVA exhibited two distinct peaks at around 320 °C and 340 °C. In contrast, PEG derivatives displayed DTGA maxima within the range of 340–410 °C, depending on their chain length. Although this overlap limits quantitative assessment of PEGylation by TGA, the appearance of a broad degradation shoulder and its chain length-dependent shift qualitatively support the successful PEGylation of the PLGA NPs. These findings complement the ^1^H NMR-based quantification and confirm the structural integrity of the NP formulation.

### 3.3 Effect of polyvinyl alcohol (PVA) washes on PLGA NPs

PVA acts as a stabilizer during emulsification of oil and water phases,^69^ and its residual presence may affect NP properties, including size, surface charge, PFCE loading (wt%), and surface chemistry. According to the literature, residual PVA can form a hydrophilic coating on the NP surface, potentially masking functional groups and interfering with surface modifications such as PEGylation.^70^ Moreover, residual PVA has been shown to impair nanoparticle performance in biological environments and affect the reproducibility of particle properties in biomedical applications.^71,72^ Furthermore, residual PVA contaminants may compete or crosslink with PEG-amine for surface access or potentially form crosslinks, thereby limiting the degree of successful carbodiimide chemistry. To address this issue, multi-step PVA washing is widely applied to reduce unbound stabilizer content and potentially enhance surface reactivity.^70^

**Scheme 2:**
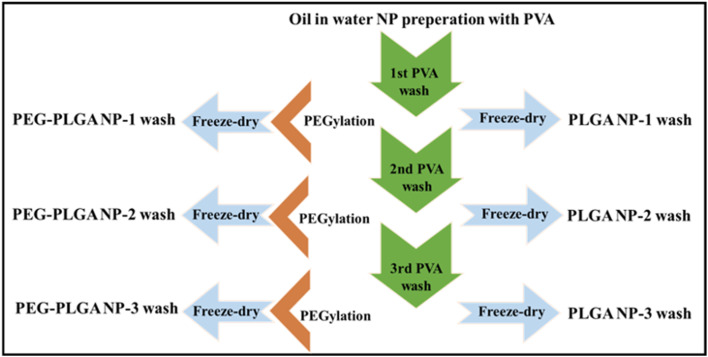
Process flow for multi-step PVA washing of PLGA NPs and PEG-PLGA NPs.

In this study, PLGA NPs with encapsulated PFCE were washed with 1, 2, or 3 cycles (centrifugation, removal of supernatant, and resuspension in distilled water). Scheme 2 outlines two experimental branches designed to explore the influence of the number of PVA washing cycles and the lyophilization step on PLGA NPs and PEG-PLGA NPs properties with PFCE entrapment. In the left branch, PLGA NPs in suspension (without prior lyophilization) were first subjected to PEGylation with the desired PEG length to assess how PEG length affects the interaction between surface-cleansed formulations. These PEGylated PLGA NPs were then lyophilized, generating PEG-PLGA NPs-# of PVA washes, where # indicates the number of PVA washing cycles. In the right branch, PLGA NPs-in suspension were directly lyophilized without PEGylation, resulting in PLGA NPs-# of PVA washes. This comparative experimental design allows a systematic evaluation of how residual PVA and lyophilization cooperatively impact downstream surface modification and PFCE loading (wt%).

Lyophilization, a commonly applied strategy to enhance the long-term shelf-life stability of NP formulations, removes water through sublimation under vacuum and low temperature.^73,74^ Despite its stabilizing benefits, lyophilization may also introduce mechanical stress during freezing and drying, potentially leading to NP aggregation, matrix deformation, or leakage of encapsulated agents such as PFCE.^75,76^ During the PEGylation optimization studies, it was observed that the number of lyophilization steps significantly influenced PFCE loading. PLGA NPs-in suspension that were PEGylated directly (without prior lyophilization), retained 12 wt% PFCE. In comparison, PLGA NPs in the solid (lyophilized) state that underwent one lyophilization cycle before PEGylation showed only 5 wt% PFCE. These findings suggest that reducing freeze-drying cycles can enhance PFCE loading and should be considered alongside PVA removal strategies when optimizing surface modification protocols.

Therefore, lyophilization is a critical step in preparing NP formulations for enhanced long-term stability and storage. However, in the absence of PEGylation, these systems may be more susceptible to PFCE loss due to the lack of a protective surface layer during processing. Incorporating PEG chains (surface PEGylation) prior to freeze-drying may contribute to improved colloidal stability, potentially by modifying the NP surface environment. PEGylation not only improves colloidal stability but can also help retain hydrophobic cargo such as PFCE possibly by providing a steric, hydrophilic shield that reduces leakage under freeze-drying stress^77,78^

The influence of PEGylation became particularly evident when comparing PFCE loading at identical PVA washing conditions. Table 1 indicates that at one wash, PEG45-PLGA NPs achieved a PFCE loading of 18.2 wt%, nearly double that of their non-PEGylated counterpart, which retained only 9.2 wt%. Similarly, after two washes, PEG26-PLGA NPs reached 17.1 wt% PFCE loading, whereas their non-PEGylated counterparts showed 6.2 wt%. Even after three washes, where PVA removal was highest, PEG45-PLGA NPs maintained superior performance (11.3 wt% PFCE loading) compared to only 4.7 wt% for the non-PEGylated formulation. These side-by-side comparisons indicate that PEGylation is associated with improved PFCE retention during processing and lyophilization.^78^ Although residual PVA may contribute to temporary colloidal stabilization, the lower PFCE wt% observed in non-PEGylated formulations suggests that PVA alone is not sufficient to maintain comparable PFCE retention under the tested conditions. Moreover, the differences in PFCE wt% suggest that PEG chain length and formulation conditions may contribute to PFCE retention and NP stability. The magnitude of improvement in PFCE loading suggests a synergistic role of PEG chain length and surface hydration in maintaining NP integrity.

**Table 1:** Influence of PVA wash frequency on PFCE features of PEG26-PLGA NP and PEG45-PLGA NP derivatives. Values represent ^19^F NMR-derived PFCE wt% from one representative NP batch per condition

Compound	Fluorine loading (wt% PFCE)	
	Non-PEGylated	PEGylated
PEG26-PLGA NPs-3 washes	5.9	15.8
PEG26-PLGA NPs-2 washes	6.4	17.1
PEG26-PLGA NPs-1 washes	6.6	15.6
PEG45-PLGA NPs-3 washes	4.7	10.3
PEG45-PLGA NPs-2 washes	5.9	10.3
PEG45-PLGA NPs-1 washes	9.2	18.2

In light of these findings, PEGylation was associated with improved PFCE encapsulation performance and contributes to the overall robustness and reliability of NP formulation design, which is an important consideration for scalable NP production and long-term storage. The results also align with previous studies reporting the vulnerability of non-PEGylated NPs during lyophilization and emphasizing the role of PEG chain length in maintaining structural stability.^69,78^ Furthermore, the data set clearly suggests that reducing lyophilization steps, particularly by avoiding freeze-drying after PVA washing, may help maintain NP integrity and improves PFCE retention during EDC/NHS-mediated PEG conjugation. In addition to formulation-related benefits, minimizing lyophilization cycles reduces both processing time and operational complexity, improving the feasibility of large-scale implementation. This is especially relevant for industrial implementation, where eliminating a freeze-drying step can substantially shorten production timelines and reduce energy consumption while maintaining PFCE loading or overall formulation quality.

The PEGylation efficiency of PLGA NPs is strongly influenced by PEG chain length and the extent of PVA removal. Using ^1^H NMR spectra, quantification of PEGylation was enabled by the monodisperse PEG derivatives, which provided precise and predictable signals based on their well-defined repeating ethylene glycol units. These PEGs were validated for purity and consistency by HPLC-MS and prior NMR analysis, ensuring accurate measurements^9,58^ As seen in Table 2, PEGylation efficiency for PEG26-PLGA NPs varied minimally across washing conditions, with values of 7.9% for 1 wash (PEG26-PLGA NPs-1 wash), 7.7% for 2 washes (PEG26-PLGA NPs-2 washes), and 9.1% for 3 washes (PEG26-PLGA NPs-3 washes). These results suggest that shorter PEG chains are less affected by residual PVA, maintaining stable attachment efficiencies regardless of the number of washing cycles. The slight increase at 3 washes suggests that additional PVA removal exposes more reactive carboxylic groups on the NP surface, allowing for slightly improved PEGylation efficiency.^79^ In contrast, the PFCE loading (wt%) in these NPs showed more pronounced differences, highlighting the balance between stabilizer removal and functional group availability.^70^

**Table 2:** Influence of PVA wash frequency on %PEGylation efficiency of PEG26-PLGA NPs and PEG45-PLGA NPs derivatives. Values represent mean ± standard deviation (SD) from two independent samples (*n* ≥ 3)

Compound	PEGylation (% mol)
PEG26-PLGA NPs-3 washes	9.1 ± 2.2
PEG26-PLGA NPs-2 washes	7.7 ± 2.9
PEG26-PLGA NPs-1 washes	7.9 ± 1.4
PEG45-PLGA NPs-3 washes	65.0 ± 5.5
PEG45-PLGA NPs-2 washes	47.8 ± 1.5
PEG45-PLGA NPs-1 washes	38.1 ± 2.9

PEG45-PLGA NPs presented a strong positive correlation between the number of washing cycles and PEGylation efficiency, with each additional cycle yielding a progressively higher increase: nearly one-fourth higher at 2 washes (PEG45-PLGA NPs-2 washes) compared to 1 wash (PEG45-PLGA NPs-1 wash), and nearly double after the 3 washes (PEG45-PLGA NPs-3 washes) compared to the 1 wash (Table 2).

Based on these results, 3 washing cycles were found to yield the highest PEGylation efficiency for PEG45-PLGA NPs under the tested conditions, resulting in enhanced steric hindrance and improved surface coverage. These observations are consistent with the TGA trend (Fig. 6) that longer PEG chains provide superior thermal stabilization and may reduce leakage of encapsulated agents, as previously shown in PEGylated PLGA NPs when compared to non-PEGylated PLGA NPs.^24^ Together, the PFCE loading and PEGylation% results highlight the importance of optimizing washing conditions based on PEG chain length: PEG26 exhibited more robust performance (less sensitive to the experimental conditions), whereas PEG45 required meticulous surface preparation to maximize its advantages.

The surface charge and Z-average size (nm) of NPs are important parameters influencing biodistribution, clearance pathways, and cellular interactions. Negative zeta potentials (≤−30 mV) contribute to colloidal stability and reduced non-specific protein adsorption, while neutral to slightly positive surface charges can enhance renal clearance.^80,81^ Very small hydrodynamic radii (*R*_h_ <6 nm) favour renal filtration, while slightly larger sizes (∼100–200 nm) are typically associated with prolonged circulation and colloidal stability.^82,83^ In our study, PEGylated PLGA NPs exhibited size and surface charge values within these latest range, with both parameters being influenced by the PEG chain length, washing conditions, and terminal amine groups.

PEG26-PLGA NPs exhibited zeta potentials of −23 mV (1 wash), −19 mV (2 washes), and −14 mV (3 washes), reflecting a gradual reduction in surface negativity with increased PVA removal and improved PEGylation. Their non-PEGylated controls showed more negative zeta potentials of −29 mV (1 wash), −26 mV (2 washes), and −24 mV (3 washes), which is consistent with a greater contribution of exposed carboxylic groups in the absence of PEGylation.

PEG45-PLGA NPs displayed stable zeta potentials of −23 to −20 mV across washing conditions, suggesting that longer PEG chains may contribute to more stable surface properties under the tested conditions. Their non-PEGylated controls showed zeta potentials of −29 mV (1 wash), −30 mV (2 washes), and −33 mV (3 washes), consistent with increased exposure of carboxylic groups after PVA removal.

The *R*_h_ (nm) of PEG45-PLGA NPs remained consistent at 170 nm after 1 and 2 washes and 180 nm after 3 washes, slightly larger than the non-PEGylated controls, which may be associated with the presence of longer PEG chains at the NP surface. Although surface PEG density and steric shielding were not directly quantified, the observed differences in zeta potential and size support the role of PEG chain length in modulating NP surface properties.

These findings highlight the interplay between PEG's chain length and washing steps in defining NP surface characteristics, with the resulting size and zeta potential values falling within ranges commonly targeted for biomedical nanocarriers. Having optimized PEGylation and confirmed successful PFCE entrapment, we next evaluated the stability of these NPs.

### 3.4 Evaluation of *in vitro* serum stability in HSA

Albumin is the most abundant protein in human plasma, accounting for a major fraction of total plasma protein mass, and is frequently enriched among the earliest proteins adsorbing onto nanoparticle surfaces upon serum exposure.^84,85^ This early interaction plays a crucial role in determining the *in vivo* fate and biodistribution of nanoparticles.^86,87^ To investigate the influence of surface PEGylation on NP stability in protein-containing medium, PLGA NPs, PEG26-PLGA -NPs, and PEG45-PLGA NPs were incubated in HSA-containing medium under physiological conditions (35 mg mL^−1^ HSA in PBS, corresponding to physiological serum albumin levels).

The impact of surface PEGylation on NP stability in albumin-containing media was assessed by monitoring their PDI and size distribution (nm) by DLS over a 20 hour incubation.

**Fig. 7:**
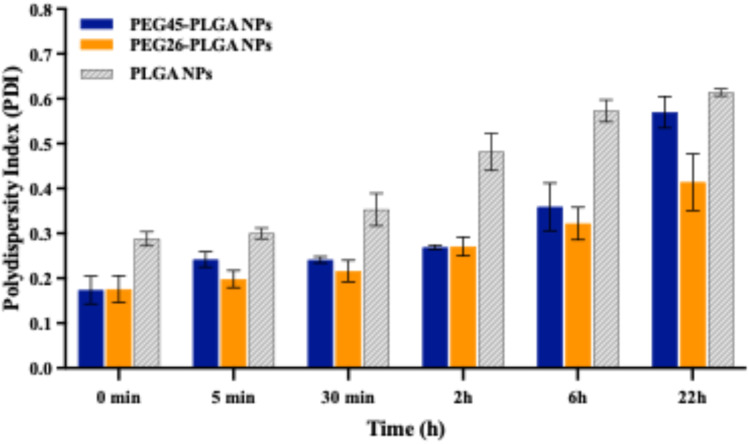
PDI values of PEG45-PLGA NPs (blue), PEG26-PLGA NPs (orange), and non-PEGylated PLGA NPs (grey) during incubation in 35 mg mL^−1^ HSA at 37 °C. Values represent mean ± standard deviation (SD) from three measurements per sample (*n* = 3).

As shown in Fig. 7, both PEG26-PLGA NPs and PEG45-PLGA NPs initially exhibited lower PDI values (∼0.2) than PLGA NPs (∼0.3). Over time, both PEGylated formulations maintained relatively low PDI values compared to non-PEGylated PLGA NPs, indicating improved colloidal stability under these conditions.

**Fig. 8:**
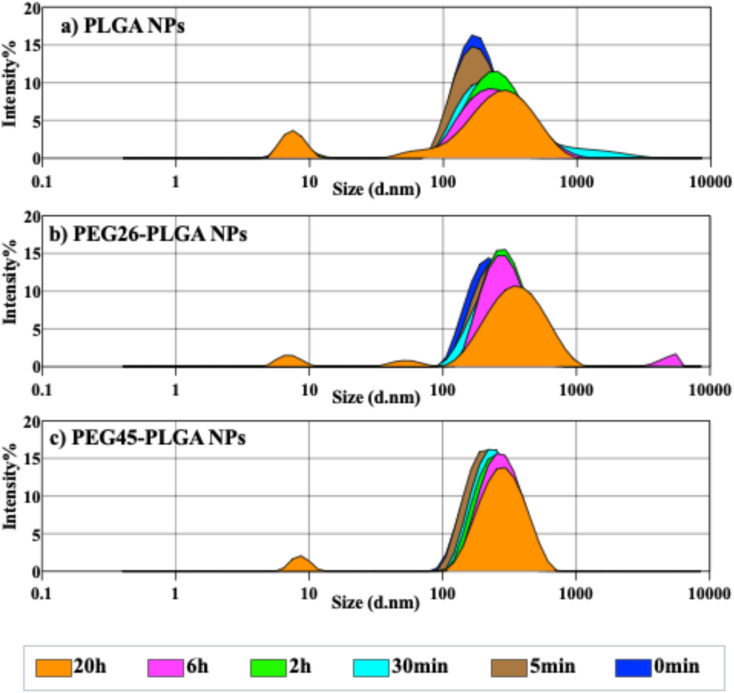
DLS Z-average size distribution (nm) overlays for the 20 h period of (a) PLGA NPs, (b) PEG26-PLGA NPs, and (c) PEG45-PLGA NPs.

In agreement with PDI trends, DLS size distributions (Fig. 8) revealed the emergence of secondary peaks in PEG26-PLGA and especially in non-PEGylated PLGA NPs over time, indicating the formation of large aggregates. These secondary peaks became particularly prominent after 6 and 20 hours in PLGA NPs (Fig. 8a), while PEG45-PLGA NPs (Fig. 8c) showed only minimal broadening of the main population and no distinct aggregation peaks. PEG26-PLGA NPs (Fig. 8b), developed an additional peak around 2000–3000 nm after prolonged incubation, consistent with lower colloidal stability than PEG45-PLGA NPs under HSA-containing conditions. This observation may be related to differences in PEG chain length and/or surface coverage, although protein adsorption and corona composition were not directly measured in the present study.

Although PEG is widely known for its anti-fouling properties, previous studies indicate that this effect depends on both PEG chain length and surface density.^88,89^ Szleifer's theoretical framework for grafted PEG surfaces predicts that protein adsorption decreases with increasing chain length and becomes largely independent of further extension once chains exceed roughly 50 ethylene oxide units (≈2200 Da), corresponding to negligible protein affinity.^90^ This behaviour is consistent with the greater stability observed for PEG45-PLGA NPs in HSA-containing medium in our study. Experimental studies with model proteins (*e.g.* lysozyme, albumin, and fibrinogen) support this model, indicating that PEG chains above ∼2200 Da achieve effective protein repulsion, while further increases in PEG molecular weight limited additional benefit. For instance, Bergström *et al.* reported no further reduction in fibrinogen adsorption on polystyrene surfaces with covalently attached PEG across the *M*_w_ range of 1500 to 20 000, suggesting a saturation threshold around 1500 Da.^91^ In addition to chain length, PEG surface density also appears critical: for nanospheres with varying PEG content (0.5–20%), optimal protein resistance was reported between 2–5% PEG, with no additional benefit at higher concentrations.^21^

Recent work has further clarified how PEGylation modulates protein corona formation and biological responses. A 2022 review on PEG in NP formulations highlights that PEG chain length, architecture, and surface density together govern protein corona composition, circulation time and immune recognition, and that PEG should be viewed as a tuneable rather than universally “stealth” coating.^92^ Systematic studies on PLGA and PLGA-PEG NPs have shown that PEGylation reduces overall protein binding and alters corona composition, including an enrichment of albumin and a depletion of opsonins, in a manner that depends on NP size but also on PEGylation efficiency.^93^ In a closely related system, PEG-PLGA NPs with comparable PEG surface presentations were recently reported to exhibit substantial PEGylation-driven remodelling of the protein corona, with distinct corona signatures arising from differences in PEG architecture and density rather than gross PEG mass alone.^28^ Our observation that PEG45-PLGA NPs display greater colloidal stability than PEG26-PLGA NPs in HSA-containing medium, despite similar overall formulation components, is consistent with these findings and suggests that relatively modest changes in PEG chain length can influence corona formation and stability. Together, these studies support the notion that well-defined PEG architectures, such as the monodisperse PEGs used here, offer a useful model for dissecting how PEG parameters shape protein corona composition, antifouling behaviour and, ultimately, the *in vivo* fate of PEG-PLGA nanocarriers.^28,94^

### 3.5 Long-term stability of post-PEGylated PLGA NPs

DLS is a key technique for NP characterization, providing data on size distributions (intensity, number, and weight), Z-average mean size, and polydispersity index (PDI), which are critical parameters for evaluating NP stability and uniformity.^95,96^ Among these parameters, Z-average is an intensity-weighted mean hydrodynamic diameter derived from cumulant analysis. Since scattered intensity scales with the sixth power of particle radius, Z-average is highly sensitive to the presence of larger particles or aggregates and can therefore overestimate the mean size in polydisperse systems.^95,96^ While this approach is effective for detecting overall size shifts, in monodisperse systems, it may also overlook low-abundance aggregates in heterogeneous systems such as PLGA NPs.^95–97^ In contrast, number-weighted distributions provide a number-normalized estimate of the average *R*_h_ for non-monodisperse samples, taking into account the contribution of each population within a broad size distribution.^98–100^ Although derived from mathematical transformation of the scattering data, number-weighted outputs can complement Z-average by making minor populations in distribution plots easier to visualize, thereby supporting a more comprehensive assessment of aggregation and size distribution.^98,101,102^ Using both Z-average and number-weighted data enables a more robust evaluation of NP stability and aggregation profile over time.^94,95,98^

In this study, both PEGylated and non-PEGylated PLGA NPs were analysed by comparing their Z-average size, number-weighted distributions, and PDI to evaluate the effects of surface modification on NP stability. Measurements were conducted in water and DMEM, at 4 °C (storage temperature), 25 °C (room temperature), and 37 °C (physiological temperature) at multiple time intervals: 0, 3, 7, 10, 17, 30, and 60 days.

**Fig. 9:**
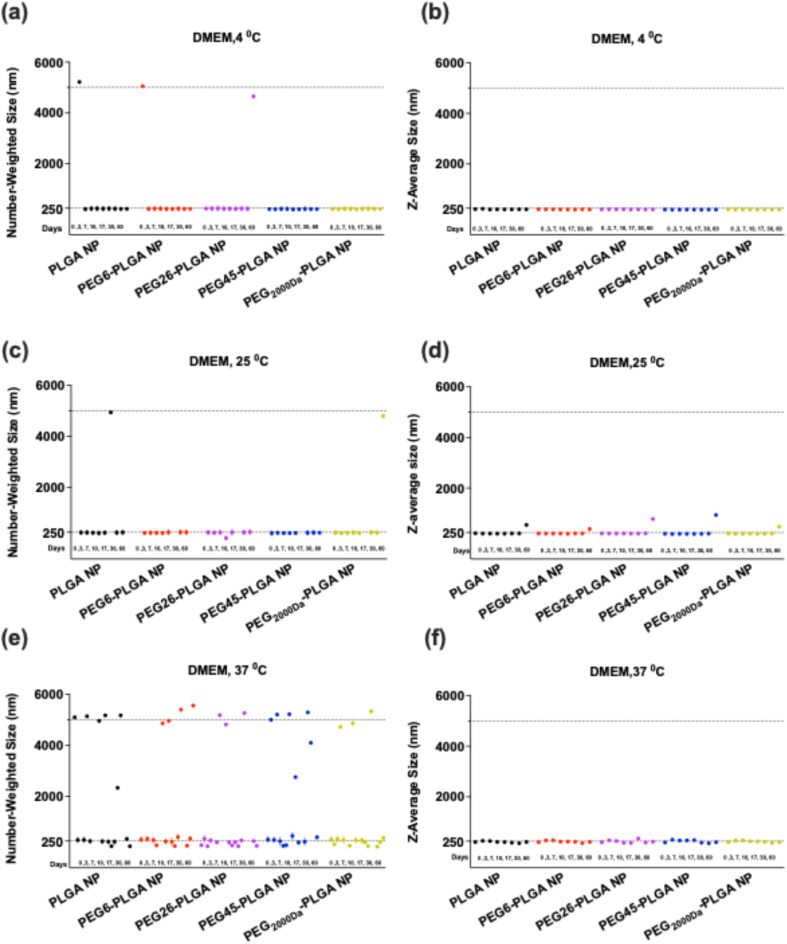
Stability results of PLGA NPs (black), PEG6-PLGA NPs (red), PEG26-PLGA NPs (purple), PEG45-PLGA NPs (blue), and PEG_2000Da_-PLGA NPs (yellow) over 60 days in DMEM, presented as number-weighted particle size (nm) at (a) 4 °C, (c) 25 °C, and (e) 37 °C, and Z-average mean size (nm) at (b) 4 °C, (d) 25 °C, and (f) 37 °C.

At 4 °C in DMEM, number-weighted size (nm) (Fig. 9a) and Z-average size (nm) (Fig. 9b) showed similar stability across most compositions. However, number-weighted distributions indicated early larger-size populations (∼5000 nm) for PLGA NPs and PEG6-PLGA NPs by day 2, which are absent in Z-average data. Additionally, PEG26-PLGA NPs showed aggregation at day 60 in the number-weighted data. Zoom-in graphs (Fig. S10) show that PEG45-PLGA NPs maintained the smallest size range (216–223 nm) over 60 days, demonstrating superior stability under cold storage conditions in DMEM (Fig. S10a).

At 25 °C in DMEM, the differences between number-weighted (Fig. 9c) and Z-average data (Fig. 9d) became more pronounced. Number-weighted data (Fig. 9c) suggest aggregation in PLGA NPs as early as day 17, with significant aggregation (∼3000 nm) (Fig. S10b). The other compositions showed comparatively stable number-weighted profiles over this period, except PEG_2000Da_-PLGA NPs, for which aggregation was observed at day 60. In contrast, Z-average data (Fig. 9d) indicated an increase in *R*_h_ (500–1000 nm) for all compositions after 30 days, including PEG45-PLGA NPs, which initially maintained the smallest *R*_h_ average size but increased to ∼1000 nm by day 60 (Fig. S10e). This increase may reflect structural rearrangements and/or the formation of low-abundance larger populations that influence the intensity-weighted mean, rather than the complete aggregation of the entire population. Notably, at day 60, aggregation in PEG_2000Da_-PLGA NPs was visible in the number-weighted distribution (Fig. 9c), with a distinct population around 2000 nm, whilethe Z-average (Fig. 9d) increased from 200 nm to 500 nm, consistent with the influence of larger particles on the intensity-weighted mean despite their low abundance. This outcome may also be influenced by uneven surface coverage resulting from PEG_2000Da_'s polydispersity and side-products, which can create localized instability.

At 37 °C, the disparity between number-weighted and Z-average data is most striking. Z-average data (Fig. 9f) indicate stability across all compositions over 60 days, with sizes consistently within the 200–300 nm range (Fig. S7f). In contrast, number-weighted distributions (Fig. 9e) reveal extensive aggregation and coarsening (∼5000 nm) across all compositions, starting as early as day 2. In albumin-containing DMEM at 25 °C and higher temperatures, protein corona formation, ionic screening, and bridging effects are known to promote NP aggregation over time, particularly when surface shielding is insufficient or heterogeneous.^103,104^

**Fig. 10:**
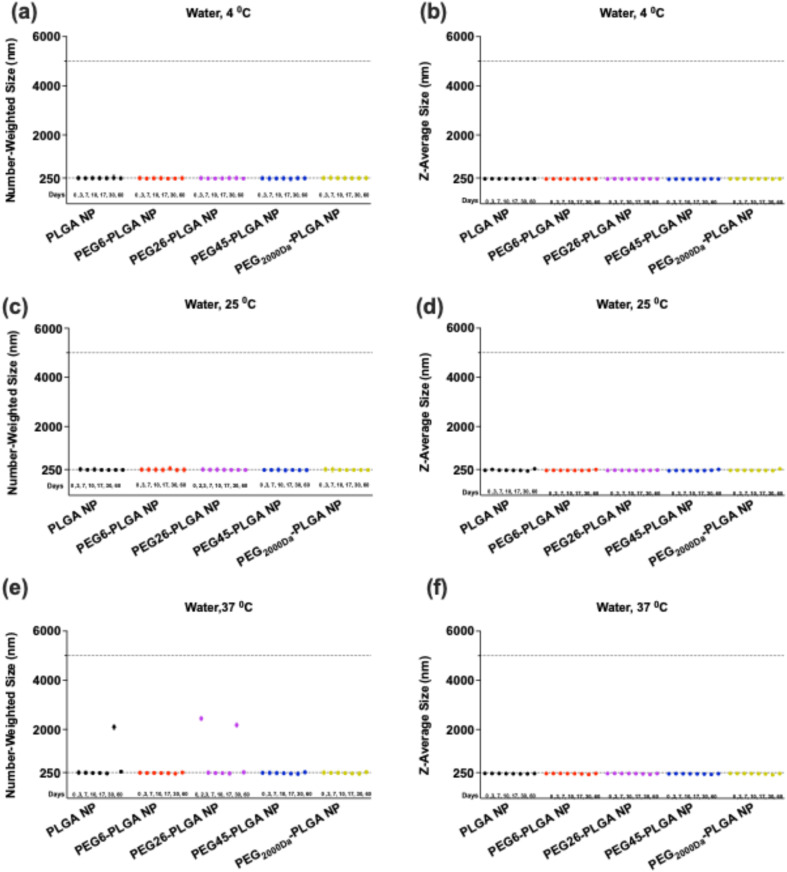
Stability results of PLGA NPs (black), PEG6-PLGA NP (red), PEG26-PLGA NPs (purple), PEG45-PLGA NPs (blue), and PEG_2000Da_-PLGA NPs (yellow) over 60 days in water, presented as number-weighted particle size (nm) data at (a) 4 °C, (c) 25 °C, and (e) 37 °C, and Z-average mean size (nm) at (b) 4 °C, (d) 25 °C, and (f) 37 °C.

At 4 °C in water, Fig. 10a and b revealed no significant differences between the two analyses, except that the number-weighted data showed (Fig. S11a) a size range approximately 50 nm larger than the Z-average (Fig. S11d), consistent with observations in DMEM.

At 25 °C in water, differences become apparent in the zoomed-in graphs. The Z-average data (Fig. S11e) show higher fluctuations in PLGA NP size over 60 days, while PEG45-PLGA NPs maintain the smallest size, suggesting comparatively better stability under this condition. By day 60, Fig. S11e revealed a particle enlargement of approximately 50 nm across all compositions, with PLGA NPs and PEG_2000Da_-PLGA NPs exhibiting the largest sizes. This size increase may reflect structural rearrangements or the gradual formation of loose aggregates, particularly in less stable formulations.

At 37 °C, the number-weighted size data (Fig. 10e) revealed larger particle populations around 2000 nm (Fig. 10f). Zoomed-in graphs (Fig. S11c and S11f) showed a slight size reduction (∼50 nm) over the first 30 days in both datasets. Deeper analysis of the number-weighted data (Fig. S11c) showed that PLGA NPs, PEG6-PLGA NPs, and PEG26-PLGA NPs exhibited larger particle populations around 2000 nm from day 17.

**Fig. 11:**
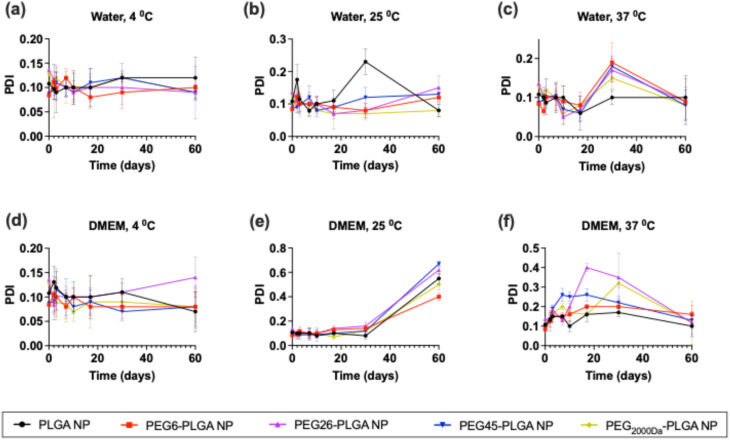
PDI results of PLGA NP (black), PEG6-PLGA NP (red), PEG26-PLGA NP (purple), PEG45-PLGA NP (blue), and PEG_2000Da_-PLGA NP (yellow) over 60 days in water, at (a) 4 °C, (b) 25 °C, and (c) 37 °C, and in DMEM at (d) 4 °C, (e) 25 °C, and (f) 37 °C.

PDI data in water at 4 °C (Fig. 11a) showed all formulations remained relatively stable over 60 days, displaying a similar profile to results in DMEM at 4 °C (Fig. 11d). At 25 °C in water (Fig. 11b), only PLGA NPs showed a notable increase in PDI at 30 days, rising from 0.1 to 0.25. In DMEM at 25 °C (Fig. 11e), all formulations exhibited a gradual PDI increase after 30 days, from 0.1 to 0.5, with PEG45-PLGA NPs and PEG26-PLGA NPs showing the highest increments, followed by PLGA NP and PEG_2000Da_-PLGA NPs.

At 37 °C, PDI values for PLGA NPs s displayed the lowest fluctuations over time in both water and DMEM (Fig. 11c and f). Overall PDI trends (Fig. 11) correlated with broadening of size distributions and the appearance of larger particle populations, particularly in PEGylated formulations at higher temperatures, underlining the importance of combining these metrics for a comprehensive assessment of NP stability.

Taken together, PEG45-PLGA NPs exhibited the most favourable stability profile under moderate storage conditions, particularly at 4 °C in DMEM and at 25 °C in water, where they maintained comparatively smaller size ranges over time. However, this improved behaviour was condition dependent. At 25 °C in DMEM, PEG45-PLGA NPs showed an increase in Z-average size by day 60, and at 37 °C in DMEM, number-weighted distributions revealed large particle populations across all formulations from early time points. Therefore, PEG45-PLGA NPs should be interpreted as enhancing colloidal stability under specific storage conditions rather than preventing aggregation under all tested conditions.

The PEG45 chain length (45 ethylene glycol units, *M*_w_: 2086, 54 Da) may contribute to the formation of a hydrophilic interfacial layer around the NP surface, which could help limit aggregation in complex media such as DMEM. This interpretation is consistent with literature describing the role of PEG in reducing protein adsorption and inter-particle interactions, particularly in protein-rich or stress-inducing environments.^17,105,106^ Additionally, the use of monodisperse PEG45 may support more reproducible surface coverage compared with polydisperse PEG_2000Da_ chains, which contain a distribution of chain lengths and may result in more heterogeneous surface properties and areas prone to instability.^17,51,52,94^

Following the optimisation of NP formulation for improved stability, the next step was to evaluate whether PEGylation induced cytotoxic effects. Biocompatibility was evaluated using Zombie-Aqua staining and flow cytometry on RAW cells and primary cells (PBMCs). PBMC cytotoxicity assays showed no detectable toxicity after 2 h exposure, with all formulations maintaining 98–100% viability relative to the negative control (Fig. S12). After 24 h, viability remained above 80% across all formulations, including PLGA NPs (82%) and PEGylated PLGA NPs (PEG6, 83%; PEG26, 81%; PEG45, 88%; PEG_2000Da_, 91%). These results suggest good preliminary biocompatibility over 24 hours, with average values above the 70–75% viability range often used as a practical cytotoxicity threshold in ISO 10993-5-based assays.^107^ In contrast, cytotoxicity assay on RAW macrophage, conducted over a 4 hour exposure period to align with the metabolic characteristics of these cells, demonstrated moderate, formulation-dependent variations in viability. For PLGA and PEGylated PLGA NPs, cell viability was as follows: PLGA NPs (65%), PEG6-PLGA NPs (69%), PEG26-PLGA NPs (57%), PEG45-PLGA NPs (71%), and PEG_2000Da_-PLGA NPs (64%). Among these, PEG45-PLGA NPs exhibited slightly higher cell viability within the RAW macrophage assay; however, overall viability values were lower and more variable than those observed in PBMCs, particularly for PEG26-PLGA NPs (Fig. S13a). These results differ from our previous data on PLGA NPs in RAW cells, where viability was consistently higher,^108–111^ and may reflect differences in PEGylation or experimental variables such as passage number and cell handling, rather than intrinsic toxicity of the PLGA core.

Additionally, the cytotoxicity of individual PEG derivatives was assessed, revealing cell viability values of 78% (PEG6), 66% (PEG26), 69% (PEG45), and 77% (PEG_2000Da_) (Fig. S13b). Taken together, these results indicate that the formulations were well tolerated in PBMCs, and that viability values in RAW macrophages were generally lower. Overall, the cytotoxicity data support the preliminary *in vitro* compatibility of the PEGylated PLGA NPs.

Under the conditions studied here, monodisperse PEG45-PLGA and conventional PEG_2000Da_-PLGA NPs showed broadly similar *in vitro* stability and cytotoxicity, with only modest, condition-dependent differences. Taken together with literature reports that PEG dispersity can influence protein adsorption and *in vivo* pharmacokinetics in other nanoparticle systems, these findings support monodisperse PEG as a rational starting point for future optimisation of PEGylated PLGA nanocarriers beyond qualitative surface modification.

We synthesized and applied monodisperse PEG-diamine derivatives as both surface ligands and quantitative internal standards, enabling precise, molar PEGylation analysis and clearer interpretation of chain-length and PVA effects that are difficult to achieve with conventional polydisperse PEGs. Their defined chain lengths are also expected to favour more homogeneous and reproducible surface coverage from batch to batch, in contrast to polydisperse PEG mixtures, where the bound chain-length distribution and effective coating composition can vary between preparations. While previous studies have reported biological advantages of monodisperse PEGs in other nanoparticle and conjugate systems, a more detailed investigation of how monodisperse *versus* polydisperse PEGylation affects the protein corona and downstream *in vivo* behaviour of PFCE-loaded PLGA NPs, including corona composition, pharmacokinetics, and biodistribution, remains an important next step^17,52^

In addition, the synthetic strategy employed here is compatible with established polymer and nanoparticle manufacturing methods, supporting future scale-up and evaluation in more complex biological models. Overall, the combination of controlled PEGylation, condition-dependent stability and acceptable short-term *in vitro* cytotoxicity on a single PFCE-loaded PLGA NP platform supports the further investigation of monodisperse PEGylated PLGA NPs as a platform for biomedical applications, while extrapolation to *in vivo* behaviour will require dedicated studies.

## 4. Conclusion

This study presents optimized strategies to improve the reproducibility and long-term stability of PEGylated PLGA NPs compared with non-PEGylated NPs. Performance was evaluated using complementary analytical and physicochemical characterizations, as well as *in vitro* cytotoxicity assessments, across PEG derivatives of different molecular weights.

We implemented and validated a scalable strategy to synthesise and purify monodisperse PEG-diamine derivatives and integrated it into a workflow that combines preparative monodisperse PEG synthesis with quantitative ^1^H NMR analysis to provide a chemically grounded assessment of PEGylation on PFCE-loaded PLGA NPs. By synthesizing PEG6-, PEG26-, and PEG45-diamine at well-defined chain lengths and validating their oligomeric purity by HPLC-MS and ^1^H NMR, we generated internal standards that overcame the analytical limitations associated with commercial polydisperse PEG_2000Da_-diamine. It should be noted that the comparison between monodisperse PEG45 and commercial polydisperse PEG_2000Da_ reflects differences in chain-length dispersity and the presence of higher-order byproducts (such as dimeric species observed by HPLC-MS), beyond the matched nominal molecular weight; this should be considered when interpreting differences in PEGylation efficiency and stability between these two systems. Using these monodisperse PEGs, we quantified PEGylation efficiencies across multiple independently prepared batches and showed that surface conjugation yields of approximately 10–13% for PEG6- and PEG45-PLGA NPs, and 19 ± 3.4% for PEG_2000Da_-PLGA NPs, can be reproducibly achieved after extensive purification. Systematic variation of PVA washing protocols revealed that residual stabilizer is a critical parameter: for PEG45-PLGA NPs, PEGylation efficiency increased from ∼38% after a single wash to ∼65% after three washes, whereas PEG26-PLGA NPs showed more modest changes. These findings highlight that both PEG chain length and PVA removal jointly determine the attainable surface PEG density. Complementary TGA/DTGA measurements further confirmed chain-length-dependent thermal stability of the PEG derivatives and qualitatively supported successful PEGylation of PLGA NPs. PFCE encapsulation efficiencies in PEGylated formulations reached up to ∼18.2%, indicating improved retention of the fluorinated cargo during the chosen lyophilization and washing workflow.

In an albumin-containing medium mimicking physiological protein level, PEG26- and PEG45-PLGA NPs maintained narrower size distributions and low PDI over 20 hours compared with non-PEGylated PLGA NPs, indicating improved colloidal stability. Over 60 days, stability was strongly dependent on storage medium and temperature: PEG45-PLGA NPs showed the smallest sizes in DMEM at 4 °C, whereas all formulations eventually aggregated in DMEM and water at 25 °C and 37 °C, particularly the PEG_2000Da_-PLGA NPs. Together, these findings show that PEG chain length, dispersity, residual PVA, and environmental conditions jointly determine colloidal stability.

Preliminary cytotoxicity assays indicated good short-term *in vitro* compatibility. In PBMCs, all nanoparticle formulations preserved near-baseline viability at 2 hours and remained above ∼80% after 24 h, with PEG45- and PEG_2000Da_-PLGA NPs giving the highest values. RAW macrophages showed a more formulation-dependent response, with viabilities of roughly 57–71% after 4 hours for PEGylated PLGA NPs and PEG45-PLGA NPs again performing at the upper end of this range. Together, these results highlight that monodisperse PEG engineering, combined with quantitative NMR analysis and controlled PVA removal, provides a level of precision that can support robust quantification of PEGylation on PLGA NPs. While the present work focuses on PFCE-loaded systems and *in vitro* read-outs, the methodology and structure–function insights established here provide a foundation for rationally designing PEGylated polymeric nanocarriers with better-defined surface chemistry and more predictable behaviour in complex biological environments, which will need to be further validated *in vivo.*

## Conflicts of interest

There are no conflicts of interest to declare.

## Supplementary Material

NA-OLF-D6NA00116E-s001

## Data Availability

The data supporting this article have been included as part of the supplementary information (SI). Supplementary information: additional characterization and stability data, including HPLC-MS and ^1^H NMR spectra of synthesized PEG derivatives and PEG‑PLGA nanoparticles, thermogravimetric analysis (TGA/DTG) profiles, extended DLS stability measurements in DMEM and water, and PBMC/RAW macrophage viability data for all formulations. See DOI: https://doi.org/10.1039/d6na00116e.

## References

[cit1] Bento C., Katz M., Santos M. M. M., Afonso C. A. M. (2024). Striving for Uniformity: A Review on Advances and Challenges To Achieve Uniform Polyethylene Glycol. Org. Process Res. Dev..

[cit2] D’souza A. A., Shegokar R. (2016). Polyethylene glycol (PEG): a versatile polymer for pharmaceutical applications. Expert Opin. Drug Deliv..

[cit3] Hoffman A. S. (2008). The origins and evolution of ‘controlled’ drug delivery systems. J. Contr. Release.

[cit4] Abuchowski A., McCoy J. R., Palczuk N. C., van Es T., Davis F. F. (1977). Effect of covalent attachment of polyethylene glycol on immunogenicity and circulating life of bovine liver catalase. J. Biol. Chem..

[cit5] Abuchowski A., van Es T., Palczuk N. C., Davis F. F. (1977). Alteration of immunological properties of bovine serum albumin by covalent attachment of polyethylene glycol. J. Biol. Chem..

[cit6] Taghavimandi F., Kim M. G., Lee M., Shin K. (2025). Beyond PEGylation: nanoparticle surface modulation for enhanced cancer therapy. Health Nanotechnol..

[cit7] Gao Y., Joshi M., Zhao Z., Mitragotri S. (2024). PEGylated therapeutics in the clinic. Bioeng. Transl. Med..

[cit8] Miao G. (2023). *et al.*, Accelerated blood clearance of PEGylated nanoparticles induced by PEG-based pharmaceutical excipients. J. Contr. Release.

[cit9] Harris J. M., Chess R. B. (2003). Effect of pegylation on pharmaceuticals. Nat. Rev. Drug Discov..

[cit10] Knop K., Hoogenboom R., Fischer D., Schubert U. S. (2010). Poly(ethylene glycol) in drug delivery: pros and cons as well as potential alternatives. Angew Chem. Int. Ed. Engl..

[cit11] Piehler J., Brecht A., Valiokas R., Liedberg B., Gauglitz G. (2000). A high-density poly(ethylene glycol) polymer brush for immobilization on glass-type surfaces. Biosens. Bioelectron..

[cit12] Veronese F. M., Pasut G. (2005). PEGylation, successful approach to drug delivery. Drug Discov. Today.

[cit13] Zhang H. (2015). *et al.*, Highly Efficient Synthesis of Monodisperse Poly(ethylene glycols) and Derivatives through Macrocyclization of Oligo(ethylene glycols). Angew. Chem..

[cit14] Siegwart D. J., Oh J. K., Matyjaszewski K. (2012). ATRP in the design of functional materials for biomedical applications. Prog. Polym. Sci..

[cit15] Mikesell L., Eriyagama D. N. A. M., Yin Y., Lu B.-Y., Fang S. (2021). Stepwise PEG synthesis featuring deprotection and coupling in one pot. Beilstein J. Org. Chem..

[cit16] Agner E. , Method for Displacement Chromatography, 2003

[cit17] Tian X., Yuan Y. (2024). Impacts of polyethylene glycol (PEG) dispersity on protein adsorption, pharmacokinetics, and biodistribution of PEGylated gold nanoparticles. RSC Adv..

[cit18] Hu J., Liu S. (2022). Emerging trends of discrete Poly(ethylene glycol) in biomedical applications. Curr. Opin. Biomed. Eng..

[cit19] Cen J., Hou M., Liu S. (2023). Discrete polyethylene glycol derivatives as a potent impetus for next-generation biomedicines. Giant.

[cit20] Suk J. S., Xu Q., Kim N., Hanes J., Ensign L. M. (2016). PEGylation as a strategy for improving nanoparticle-based drug and gene delivery. Adv. Drug Deliv. Rev..

[cit21] Gref R. (2000). *et al.*, Stealth’ corona-core nanoparticles surface modified by polyethylene glycol (PEG): influences of the corona (PEG chain length and surface density) and of the core composition on phagocytic uptake and plasma protein adsorption. Colloids Surf. B Biointerfaces.

[cit22] Makadia H. K., Siegel S. J. (2011). Poly Lactic-co-Glycolic Acid (PLGA) as Biodegradable Controlled Drug Delivery Carrier. Polymers.

[cit23] Alsaab H. O. (2022). *et al.*, PLGA-Based Nanomedicine: History of Advancement and Development in Clinical Applications of Multiple Diseases. Pharmaceutics.

[cit24] Sheffey V. V., Siew E. B., Tanner E. E. L., Eniola-Adefeso O. (2022). PLGA's Plight and the Role of Stealth Surface Modification Strategies in Its Use for Intravenous Particulate Drug Delivery. Adv. Healthcare Mater..

[cit25] El-Hammadi M. M., Arias J. L. (2022). Recent Advances in the Surface Functionalization of PLGA-Based Nanomedicines. Nanomaterials.

[cit26] Kesharwani P. (2025). *et al.*, PEGylated PLGA nanoparticles: unlocking advanced strategies for cancer therapy. Mol. Cancer.

[cit27] Dodda J. M., Remiš T., Rotimi S., Yeh Y.-C. (2022). Progress in the drug encapsulation of poly(lactic-co-glycolic acid) and folate-decorated poly(ethylene glycol)–poly(lactic-co-glycolic acid) conjugates for selective cancer treatment. J. Mater. Chem. B.

[cit28] Spinelli L. (2025). *et al.*, PEGylation-Driven Remodeling of the Protein Corona on PLGA Nanoparticles: Implications for Macrophage Recognition. Biomacromolecules.

[cit29] Spek S., Haeuser M., Schaefer M. M., Langer K. (2015). Characterisation of PEGylated PLGA nanoparticles comparing the nanoparticle bulk to the particle surface using UV/vis spectroscopy, SEC, 1H NMR spectroscopy, and X-ray photoelectron spectroscopy. Appl. Surf. Sci..

[cit30] D'Antone S. (2001). *et al.*, Thermogravimetric investigation of two classes of block copolymers based on poly(lactic-glycolic acid) and poly(ε-caprolactone) or poly(ethylene glycol). Polym. Degrad. Stab..

[cit31] Betancourt T. (2009). *et al.*, PEGylation strategies for active targeting of PLA/PLGA nanoparticles. J. Biomed. Mater. Res., Part A.

[cit32] Grabarek Z., Gergely J. (1990). Zero-length crosslinking procedure with the use of active esters. Anal. Biochem..

[cit33] Essa D., Choonara Y. E., Kondiah P. P. D., Pillay V. (2020). Comparative Nanofabrication of PLGA-Chitosan-PEG Systems Employing Microfluidics and Emulsification Solvent Evaporation Techniques. Polymers.

[cit34] Srinivas M. (2010). *et al.*, Customizable, multi-functional fluorocarbon nanoparticles for quantitative in vivo imaging using 19F MRI and optical imaging. Biomaterials.

[cit35] Swider E. (2018). *et al.*, Design of triphasic poly(lactic-co-glycolic acid) nanoparticles containing a perfluorocarbon phase for biomedical applications. RSC Adv..

[cit36] Koshkina O. (2020). *et al.*, Nanoparticles for “two color” 19F magnetic resonance imaging: Towards combined imaging of biodistribution and degradation. J. Colloid Interface Sci..

[cit37] Krafft M. P., Riess J. G. (2009). Chemistry, Physical Chemistry, and Uses of Molecular Fluorocarbon−Hydrocarbon Diblocks, Triblocks, and Related Compounds—Unique “Apolar” Components for Self-Assembled Colloid and Interface Engineering. Chem. Rev..

[cit38] Hartmann D., Greb L. (2020). [Si(O2C6F4)2]14: Self-Assembly of a Giant Perfluorinated Macrocyclic Host by Low-Barrier Si−O Bond Metathesis. Angew. Chem., Int. Ed..

[cit39] Kirsch P. , Introduction of Fluorine, in Modern Fluoroorganic Chemistry, ed. P. Kirsch, John Wiley & Sons, Ltd, 2013, pp. 25–106, 10.1002/9783527651351.ch2

[cit40] Ahrens E. T., Flores R., Xu H., Morel P. A. (2005). In vivo imaging platform for tracking immunotherapeutic cells. Nat. Biotechnol..

[cit41] Mali A. (2025). *et al.*, Polymeric (Poly(lactic-co-glycolic acid)) Particles Entrapping Perfluorocarbons Are Stable for a Minimum of Six Years. ACS Omega.

[cit42] Cruz L. J. (2010). *et al.*, Targeted PLGA nano- but not microparticles specifically deliver antigen to human dendritic cells via DC-SIGN in vitro. J. Controlled Release.

[cit43] Böyum A. (1968). Isolation of mononuclear cells and granulocytes from human blood. Isolation of monuclear cells by one centrifugation, and of granulocytes by combining centrifugation and sedimentation at 1 g. Scand. J. Clin. Lab. Invest., Suppl..

[cit44] Zhang L. (2025). *et al.*, Role of PEGylated lipid in lipid nanoparticle formulation for in vitro and in vivo delivery of mRNA
vaccines. J. Controlled Release.

[cit45] Mishra P., Nayak B., Dey R. K. (2016). PEGylation in anti-cancer therapy: An overview. Asian J. Pharm. Sci..

[cit46] Giese M. W. , Woodman R. H., Hermanson G. T. and Davis P. D., The Use of Uniform PEG Compounds in the Design of ADCs, 2021, 10.1039/9781839165153-00286

[cit47] Richter A. W., Akerblom E. (1984). Polyethylene glycol reactive antibodies in man: titer distribution in allergic patients treated with monomethoxy polyethylene glycol modified allergens or placebo, and in healthy blood donors. Int. Arch. Allergy Appl. Immunol..

[cit48] Yang Q. (2016). *et al.*, Analysis of Pre-existing IgG and IgM Antibodies against Polyethylene Glycol (PEG) in the General Population. Anal. Chem..

[cit49] Fu J., Wu E., Li G., Wang B., Zhan C. (2024). Anti-PEG antibodies: Current situation and countermeasures. Nano Today.

[cit50] McSweeney M. D., Mohan M., Commins S. P., Lai S. K. (2021). Anaphylaxis to Pfizer/BioNTech mRNA COVID-19 Vaccine in a Patient With Clinically Confirmed PEG Allergy. Front. Allergy.

[cit51] Zhang P., Zhang Z., Wang D., Hao J., Cui J. (2020). Monodispersity of Poly(ethylene glycol) Matters for Low-Fouling Coatings. ACS Macro Lett..

[cit52] Wang J. (2020). *et al.*, Monodisperse and Polydisperse PEGylation of Peptides and Proteins: A Comparative Study. Biomacromolecules.

[cit53] Keegstra E. M. D., Zwikker J. W., Roest M. R., Jenneskens L. W. (1992). A highly selective synthesis of monodisperse oligo(ethylene glycols). J. Org. Chem..

[cit54] Khanal A., Fang S. (2017). Solid Phase Stepwise Synthesis of Polyethylene Glycols. Chem.–Eur. J..

[cit55] Wawro A. M., Muraoka T., Kato M., Kinbara K. (2016). Multigram chromatography-free synthesis of octa(ethylene glycol) p-toluenesulfonate. Org. Chem. Front..

[cit56] Zhu J. (2019). *et al.*, Peptidic Monodisperse PEG “combs” with Fine-Tunable LCST and Multiple Imaging Modalities. Biomacromolecules.

[cit57] Ait Bachir Z. (2018). *et al.*, Effects of PEG surface density and chain length on the pharmacokinetics and biodistribution of methotrexate-loaded chitosan nanoparticles. Int. J. Nanomed..

[cit58] Pozzi D. (2014). *et al.*, Effect of polyethyleneglycol (PEG) chain length on the bio-nano-interactions between PEGylated lipid nanoparticles and biological fluids: from nanostructure to uptake in cancer cells. Nanoscale.

[cit59] Wang J.-L. (2018). *et al.*, The effect of surface poly(ethylene glycol) length on in vivo drug delivery behaviors of polymeric nanoparticles. Biomaterials.

[cit60] Sun J., Walker J., Beck-Broichsitter M., Schwendeman S. P. (2022). Characterization of commercial PLGAs by NMR spectroscopy. Drug Deliv. Transl. Res..

[cit61] Bijarimi M., Ahmad S., Rasid R., Khushairi M. A., Zakir M. (2016). Poly(lactic acid)/Poly(ethylene glycol) blends: Mechanical, thermal and morphological properties. AIP Conf. Proc..

[cit62] Douglas P. (2016). *et al.*, Effect of poly ethylene glycol on the mechanical and thermal properties of bioactive poly(ε-caprolactone) melt extrudates for pharmaceutical applications. Int. J. Pharm..

[cit63] Filho O. P. da S. (2021). *et al.*, A comparison of acyl-moieties for noncovalent functionalization of PLGA and PEG-PLGA nanoparticles with a cell-penetrating peptide. RSC Adv..

[cit64] Lopez-Mitjavila J. J. (2025). *et al.*, PEGylated PLGA nanoparticles prepared from nano-emulsion templates as versatile platforms to cross blood–brain barrier models. J. Drug Delivery Sci. Technol..

[cit65] Mushtaq A., Li L., A A., Grøndahl L. (2022). Characterisation of products from EDC-mediated PEG substitution of chitosan allows optimisation of reaction conditions. Int. J. Biol. Macromol..

[cit66] Kaldéus T., Nordenström M., Carlmark A., Wågberg L., Malmström E. (2018). Insights into the EDC-mediated PEGylation of cellulose nanofibrils and their colloidal stability. Carbohydr. Polym..

[cit67] Bhattacharya S., Shunmugam R. (2020). Unraveling the Effect of PEG Chain Length on the Physical Properties and Toxicant Removal Capacities of Cross-Linked Network Synthesized by Thiol–Norbornene Photoclick Chemistry. ACS Omega.

[cit68] Balcı M. (2010). *et al.*, Synthesis and characterization of novel comb-type amphiphilic graft copolymers containing polypropylene and polyethylene glycol. Polym. Bull..

[cit69] Song B., Cho C.-W. (2024). Applying polyvinyl alcohol to the preparation of various nanoparticles. J. Pharm. Investig..

[cit70] Sahoo S. K., Panyam J., Prabha S., Labhasetwar V. (2002). Residual polyvinyl alcohol associated with poly (d,l-lactide-co-glycolide) nanoparticles affects their physical properties and cellular uptake. J. Controlled Release.

[cit71] Yang M. (2014). *et al.*, Nanoparticle penetration of human cervicovaginal mucus: The effect of polyvinyl alcohol. J. Controlled Release.

[cit72] Feczkó T., Tóth J., Dósa G., Gyenis J. (2011). Influence of process conditions on the mean size of PLGA nanoparticles. Chem. Eng. Process. Process Intensif..

[cit73] Chen C., Han D., Cai C., Tang X. (2010). An overview of liposome lyophilization and its future potential. J. Controlled Release.

[cit74] Chen Y. (2021). *et al.*, Freeze-Drying Formulations Increased the Adenovirus and Poxvirus Vaccine Storage Times and Antigen Stabilities. Virol. Sin..

[cit75] Kommineni N., Butreddy A., Sainaga Jyothi V. G. S., Angsantikul P. (2022). Freeze-drying for the preservation of immunoengineering products. iScience.

[cit76] Wang Y., Grainger D. W. (2019). Lyophilized liposome-based parenteral drug development: Reviewing complex product design strategies and current regulatory environments. Adv. Drug Delivery Rev..

[cit77] Narváez-Narváez D. A. (2023). *et al.*, Comparative Analysis of the Physicochemical and Biological Characteristics of Freeze-Dried PEGylated Cationic Solid Lipid Nanoparticles. Pharmaceuticals.

[cit78] Gatto M. S., Najahi-Missaoui W. (2023). Lyophilization of Nanoparticles, Does It Really Work? Overview of the Current Status and Challenges. Int. J. Mol. Sci..

[cit79] Owens D. E., Peppas N. A. (2006). Opsonization, biodistribution, and pharmacokinetics of polymeric nanoparticles. Int. J. Pharm..

[cit80] Mosleh-Shirazi S., Abbasi M., Shafiee M., Kasaee S. R., Amani A. M. (2021). Renal clearable nanoparticles: An expanding horizon for improving biomedical imaging and cancer therapy. Mater. Today Commun..

[cit81] Honary S., Zahir F. (2013). Effect of Zeta Potential on the Properties of Nano-Drug Delivery Systems - A Review (Part 1). Trop. J. Pharmaceut. Res..

[cit82] Blanco E., Shen H., Ferrari M. (2015). Principles of nanoparticle design for overcoming biological barriers to drug delivery. Nat. Biotechnol..

[cit83] Soo Choi H. (2007). *et al.*, Renal clearance of quantum dots. Nat. Biotechnol..

[cit84] Qu N. (2024). *et al.*, Albumin Nanoparticle-Based Drug Delivery Systems. Int. J. Nanomed..

[cit85] Vincent M. P. (2021). *et al.*, Surface chemistry-mediated modulation of adsorbed albumin folding state specifies nanocarrier clearance by distinct macrophage subsets. Nat. Commun..

[cit86] Li L., Jiang X., Gao J. (2022). Characterization and Biomedical Application Opportunities of the Nanoparticle's Protein Corona. Adv. Mater. Interfaces.

[cit87] Dobrovolskaia M. A., Aggarwal P., Hall J. B., McNeil S. E. (2008). Preclinical Studies To Understand Nanoparticle Interaction with the Immune System and Its Potential
Effects on Nanoparticle Biodistribution. Mol. Pharm..

[cit88] Nagasaki Y. (2011). Construction of a densely poly(ethylene glycol)-chain-tethered surface and its performance. Polym. J..

[cit89] Arcot L., Ogaki R., Zhang S., Meyer R. L., Kingshott P. (2015). Optimizing the surface density of polyethylene glycol chains by grafting from binary solvent mixtures. Appl. Surf. Sci..

[cit90] Szleifer I. (1997). Protein adsorption on surfaces with grafted polymers: a theoretical approach. Biophys. J..

[cit91] Bergström K., Holmberg K., Safranj A., Hoffman A. S., Edgell M. J., Kozlowski A., Hovanes B. A., Harris J. M. (1992). Reduction of fibrinogen adsorption on PEG-coated polystyrene surfaces. J. Biomed. Mater. Res..

[cit92] Padín-González E. (2022). *et al.*, Understanding the Role and Impact of Poly (Ethylene Glycol) (PEG) on Nanoparticle Formulation: Implications for COVID-19 Vaccines. Front. Bioeng. Biotechnol..

[cit93] Partikel K. (2019). *et al.*, Effect of nanoparticle size and PEGylation on the protein corona of PLGA nanoparticles. Eur. J. Pharm. Biopharm..

[cit94] Stetefeld J., McKenna S. A., Patel T. R. (2016). Dynamic light scattering: a practical guide and applications in biomedical sciences. Biophys. Rev..

[cit95] Rodriguez-Loya J., Lerma M., Gardea-Torresdey J. L. (2023). Dynamic Light Scattering and Its Application to Control Nanoparticle Aggregation in Colloidal Systems: A Review. Micromachines.

[cit96] Bhattacharjee S. (2016). DLS and zeta potential – What they are and what they are not?. J. Controlled Release.

[cit97] Jin L., Jarand C. W., Brader M. L., Reed W. F. (2022). Angle-dependent effects in DLS measurements of polydisperse particles. Meas. Sci. Technol..

[cit98] Farkas N., Kramar J. A. (2021). Dynamic Light Scattering Distributions by Any Means. J. Nanopart. Res..

[cit99] Number and volume size distributions. https://www.malvernpanalytical.com/es/learn/knowledge-center/application-notes/an140403numbervolumesizedistributions

[cit100] Yeap S. P., Lim J., Ngang H. P., Ooi B. S., Ahmad A. L. (2018). Role of Particle-Particle Interaction Towards Effective Interpretation of Z-Average and Particle Size Distributions from Dynamic Light Scattering (DLS) Analysis. J. Nanosci. Nanotechnol..

[cit101] Creese G. , Data analysis and distribution weighting. Brookhaven Instruments, https://www.brookhaveninstruments.com/data-analysis-and-distribution-weighting-faq-for-dls-users/, 2020

[cit102] Frisken B. J. (2001). Revisiting the method of cumulants for the analysis of dynamic light-scattering data. Appl. Opt..

[cit103] Park S. J. (2020). Protein–Nanoparticle Interaction: Corona Formation and Conformational Changes in Proteins on Nanoparticles. Int. J. Nanomed..

[cit104] Bashiri G. (2023). *et al.*, Nanoparticle protein corona: from structure and function to therapeutic targeting. Lab Chip.

[cit105] Bernhard C. (2017). *et al.*, Repelling and ordering: the influence of poly(ethylene glycol) on protein adsorption. Phys. Chem. Chem. Phys..

[cit106] Gooch N. W., Hlady V. (2015). Two surface gradients of polyethylene glycol for a reduction in protein adsorption. Surf. Innov..

[cit107] Gruber S., Nickel A. (2023). Toxic or not toxic? The specifications of the standard ISO 10993-5 are not explicit enough to yield comparable results in the cytotoxicity assessment of an identical medical device. Front. Med. Technol..

[cit108] Mali A. (2023). *et al.*, The internal structure of gadolinium and perfluorocarbon-loaded polymer nanoparticles affects 19 F MRI relaxation times. Nanoscale.

[cit109] Mali A. (2026). *et al.*, Nanoparticle ultrastructure allows reversible pH sensitivity using 19F NMR and in vivo19F MRI. Nanoscale Adv..

[cit110] Larreina Vicente N., Srinivas M., Tagit O. (2025). Perfluorocarbon-Loaded Poly(lactide-co-glycolide) Nanoparticles from Core to Crust: Multifaceted Impact of Surfactant on Particle Ultrastructure, Stiffness, and Cell Uptake. ACS Appl. Polym. Mater..

[cit111] Staal A. H. J. (2020). *et al.*, In vivo clearance of 19F MRI imaging nanocarriers is strongly influenced by nanoparticle ultrastructure. Biomaterials.

