# Supplementary material for: Monodisperse PEG engineering for quantifiable surface conjugation on PLGA nanoparticles
Source: Nanoscale Adv. 2026 Jun 30;8(16):4500–22. doi: 10.1039/d6na00116e (PMC13356874; doi:10.1039/d6na00116e)
Supplement: Supplementary file 1 [file NA-008-D6NA00116E-s001.pdf]

Supplementary Information (SI) for Nanoscale Advances.
This journal is © The Royal Society of Chemistry 2026

Supplementary Information to:

Monodisperse PEG Engineering for Quantifiable Surface Conjugation on PLGA Nanoparticles

Ezgi Basavci[1,2], Alvja Mali[1] , Marjan Kalati[1], Raphael Marques Marcilli[1], Mangala Srinivas[1],

[1] Cell Biology and Immunology, Wageningen University and Research (WUR), Wageningen, Netherlands
[2,] Polypure AS, Oslo, Norway

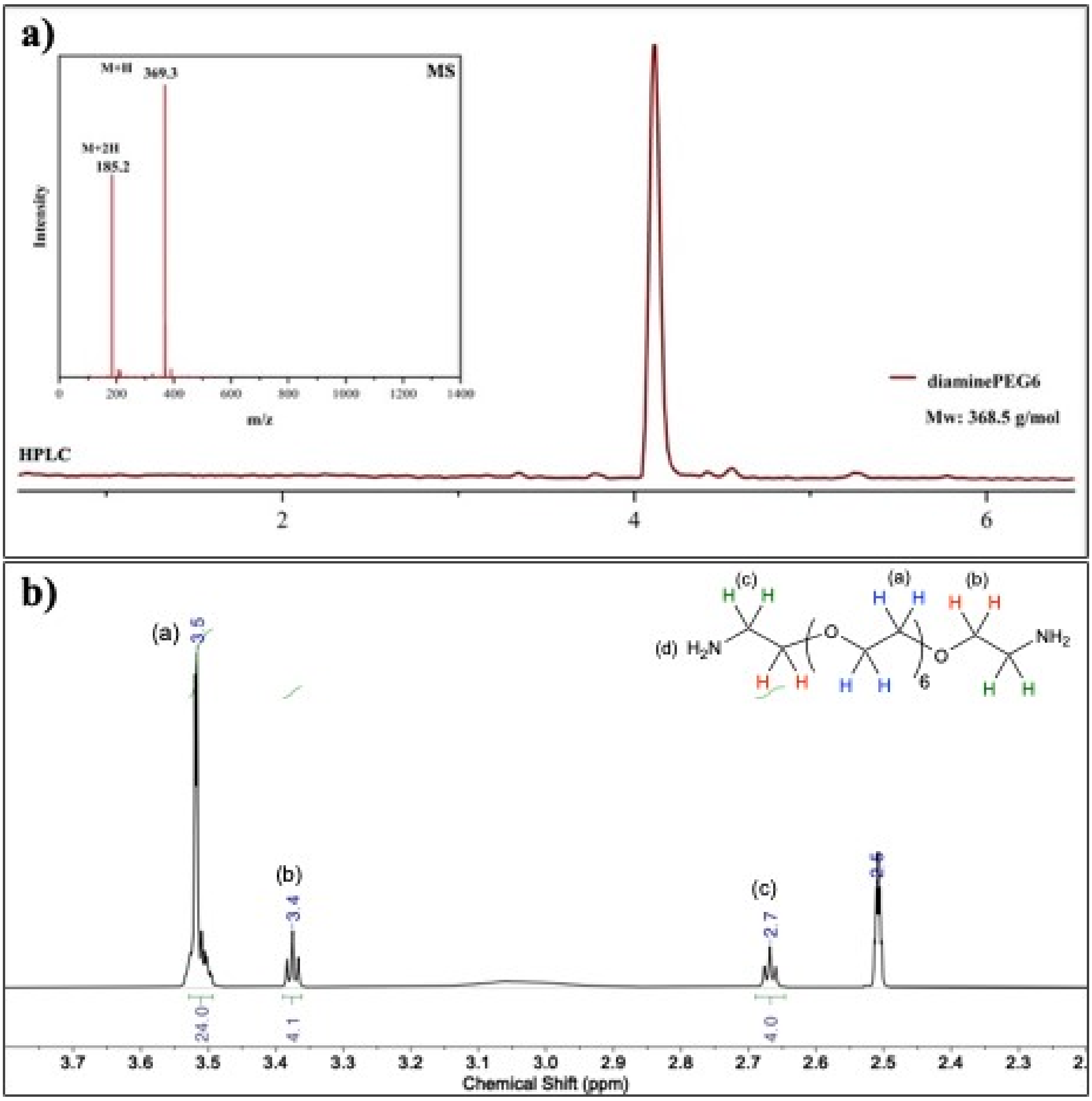

Figure S 1: (a) HPLC chromatogram and MS spectrum, and **(b)** ^{1}H NMR spectrum of synthesized monodisperse PEG6-diamine (700 MHz, DMSO-d6).

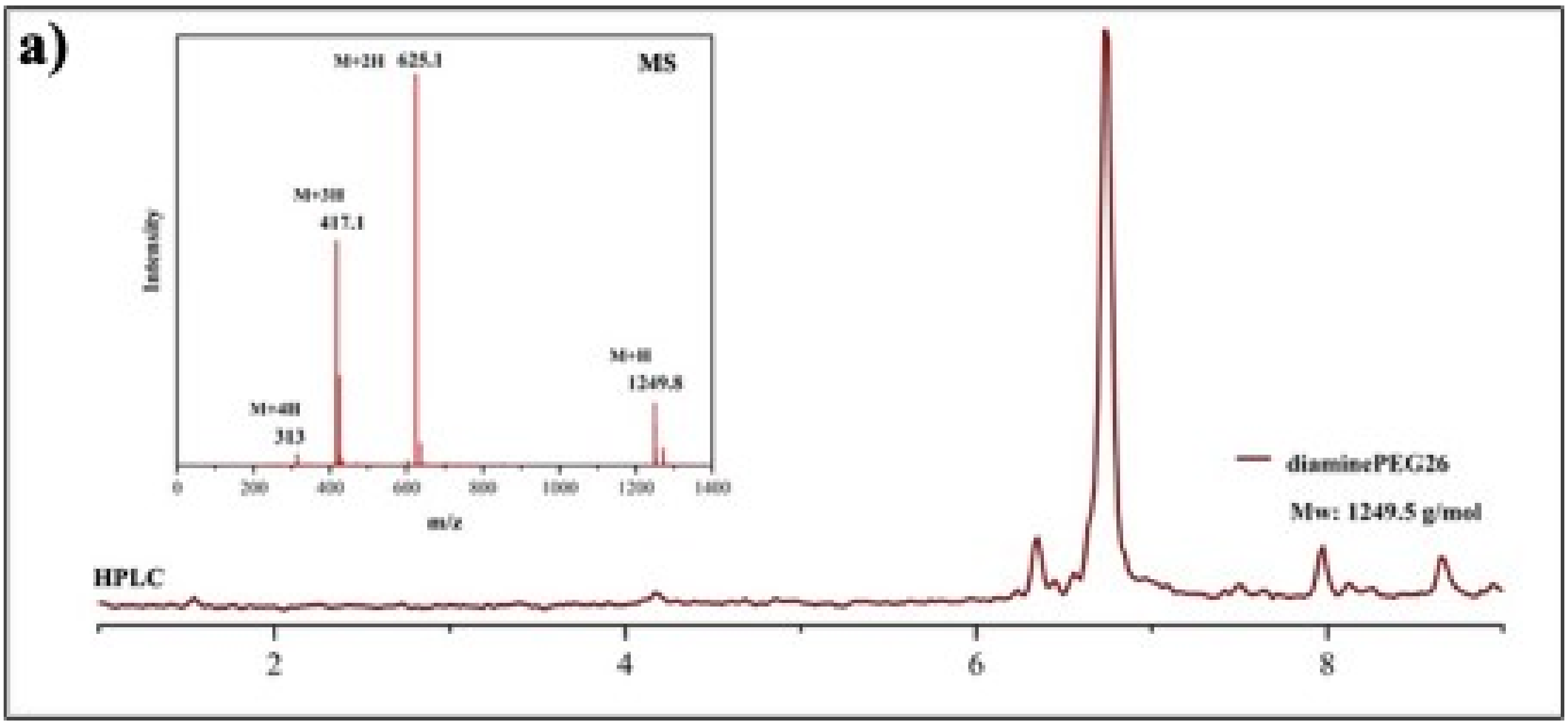

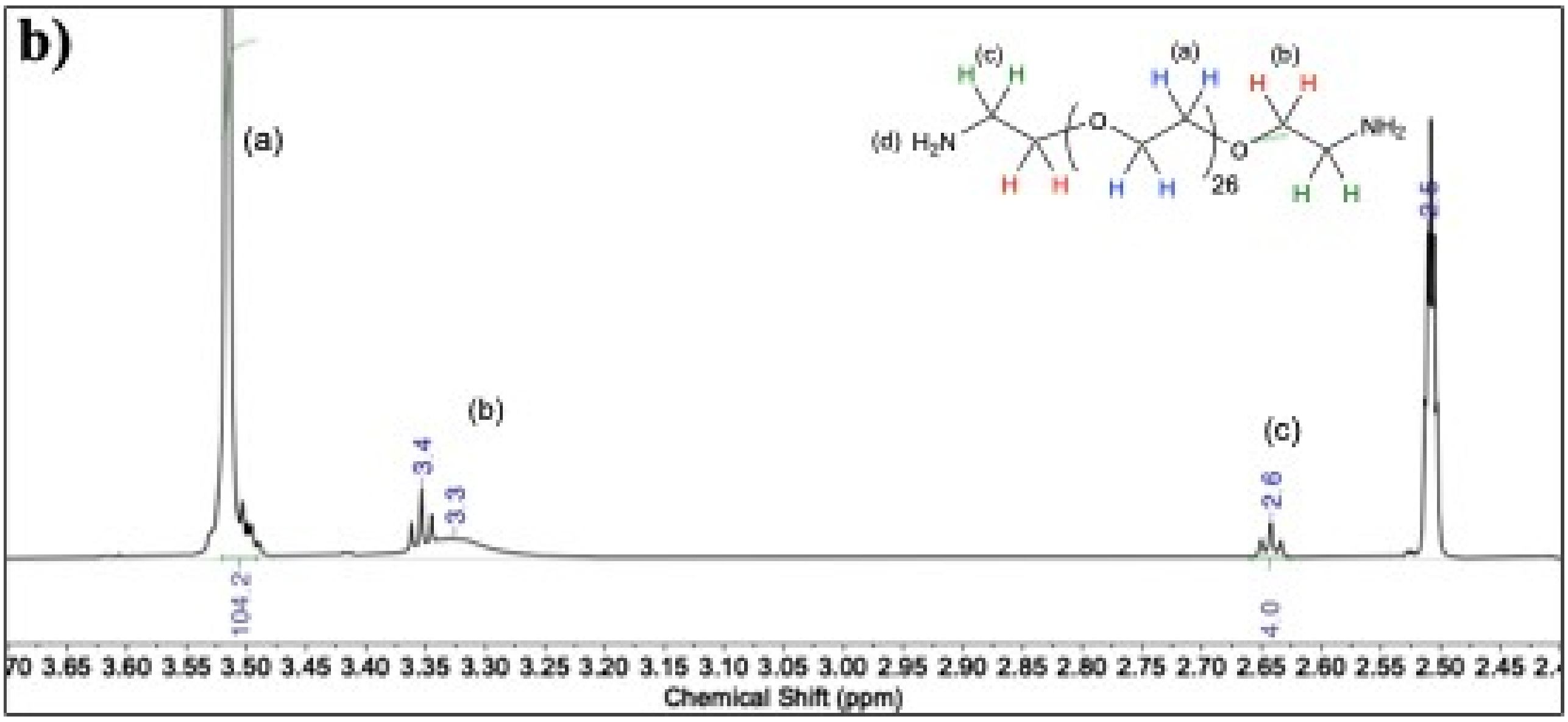

Figure S 2: (a) HPLC chromatogram and MS spectrum, and **(b)** ^{1}H NMR spectrum of synthesized monodisperse PEG26-diamine (700 MHz, DMSO-d6).

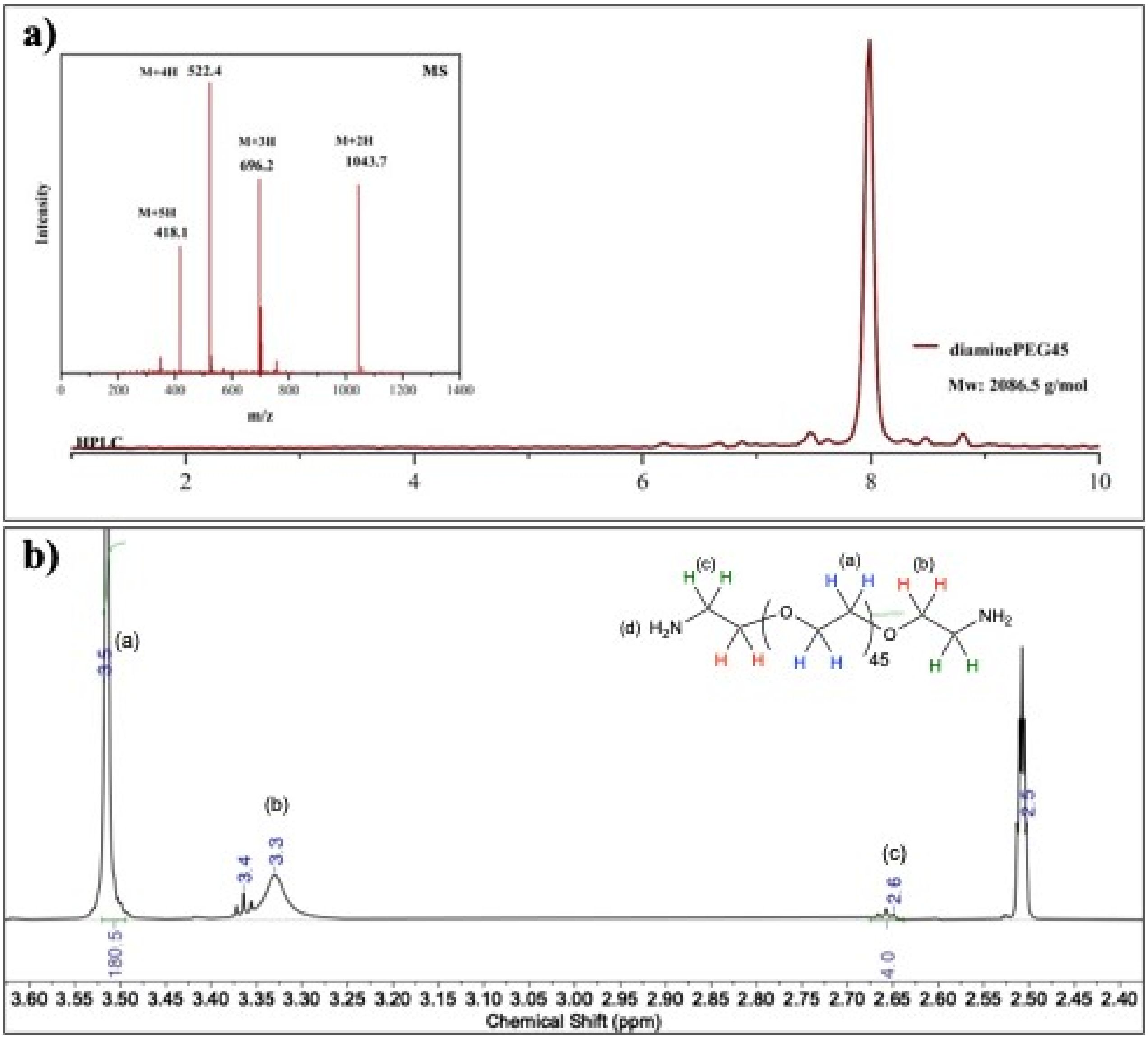

Figure S 3: (a) HPLC chromatogram and MS spectrum, and **(b)** ^{1}H NMR spectrum of synthesized monodisperse PEG45-diamine (700 MHz, DMSO-d6).

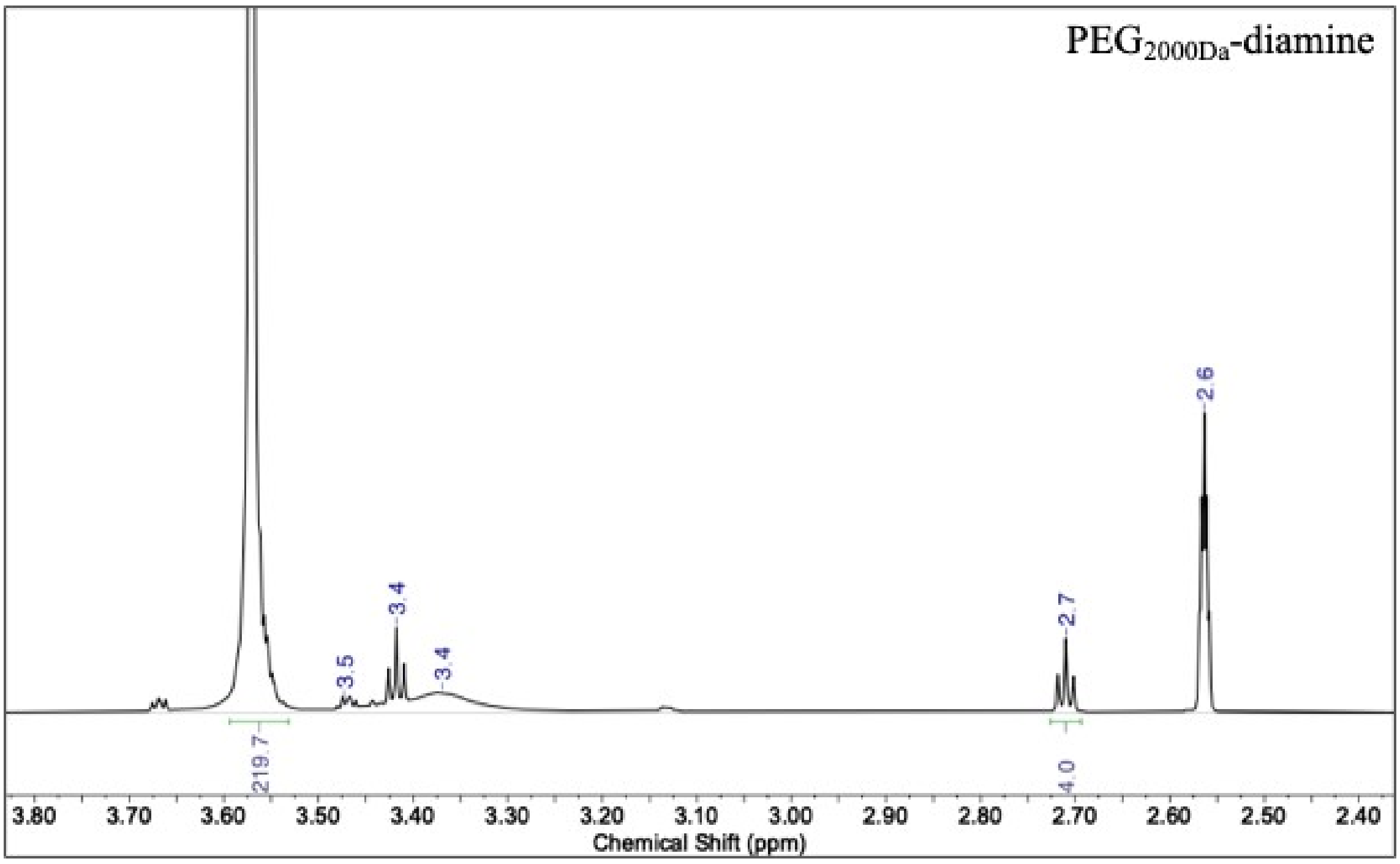

Figure S 4: ^{1}H NMR spectrum of commercial PEG_{2000Da}-diamine (700 MHz, DMSO-d6).

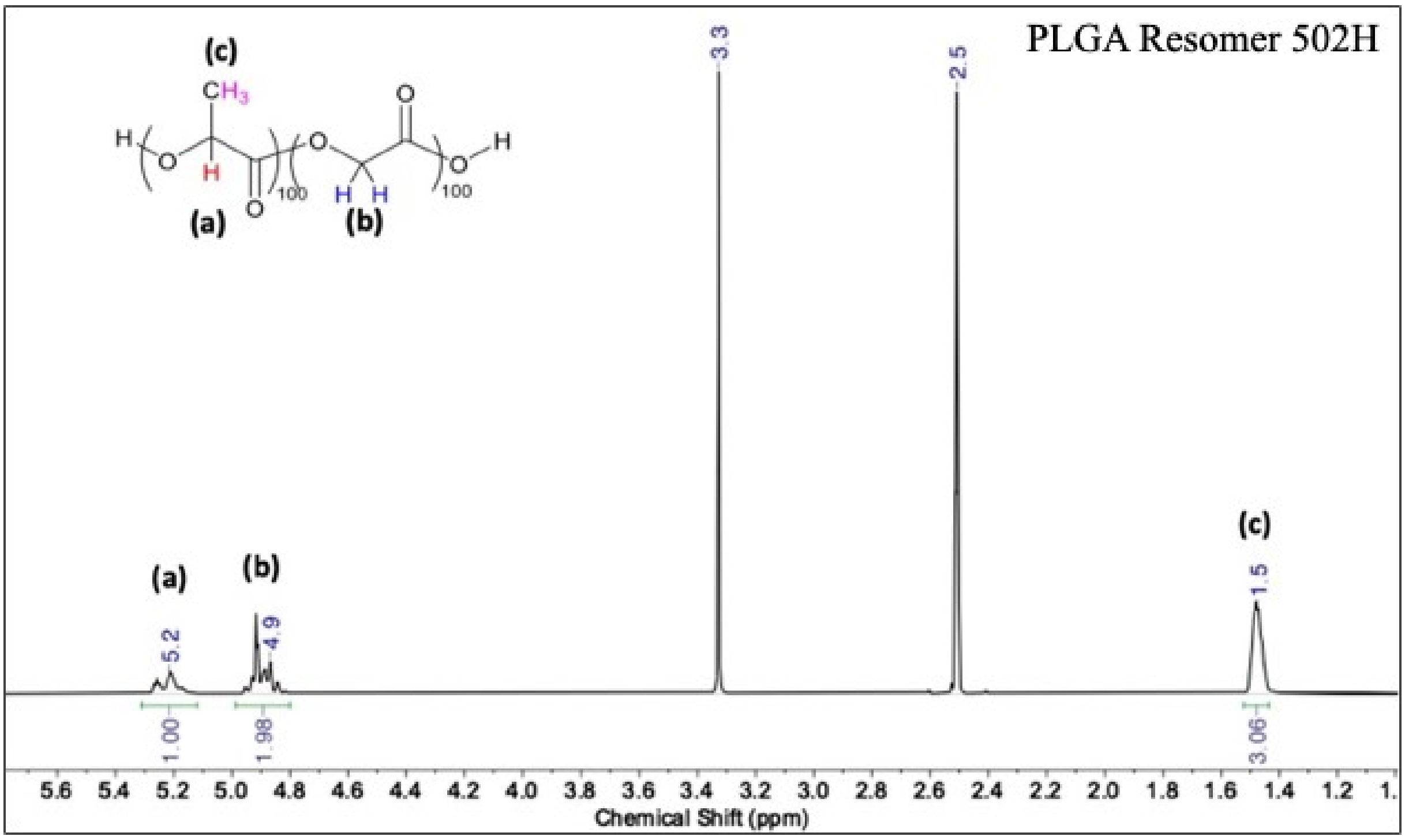

Figure S 5:^{1}H NMR spectra of commercial PLGA Resomer® RG 502H polymer (700 MHz, d6-DMSO).

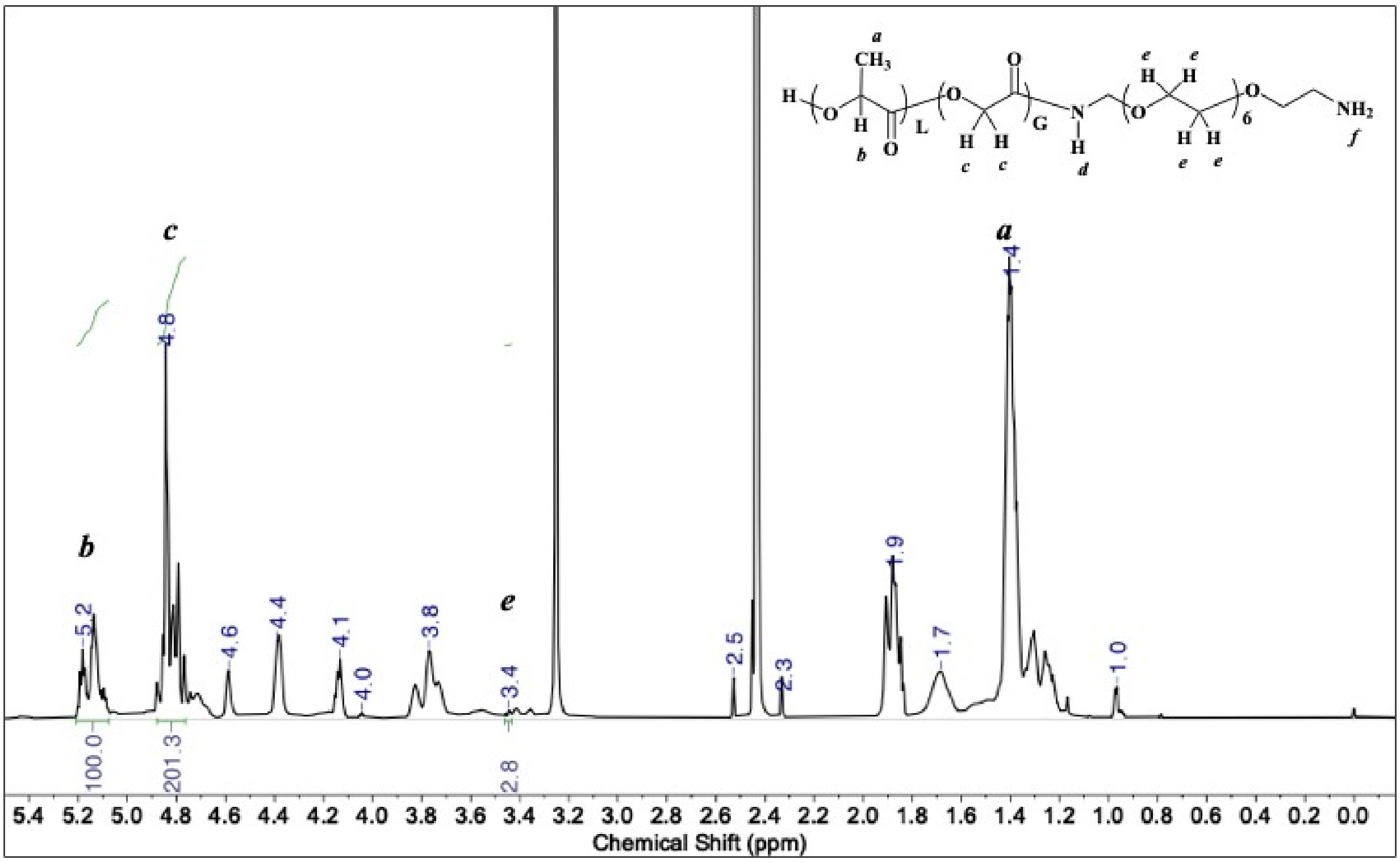

Figure S 6: ^{1}H NMR spectra of synthesized PEG6-PLGA NPs (700 MHz, d6-DMSO).

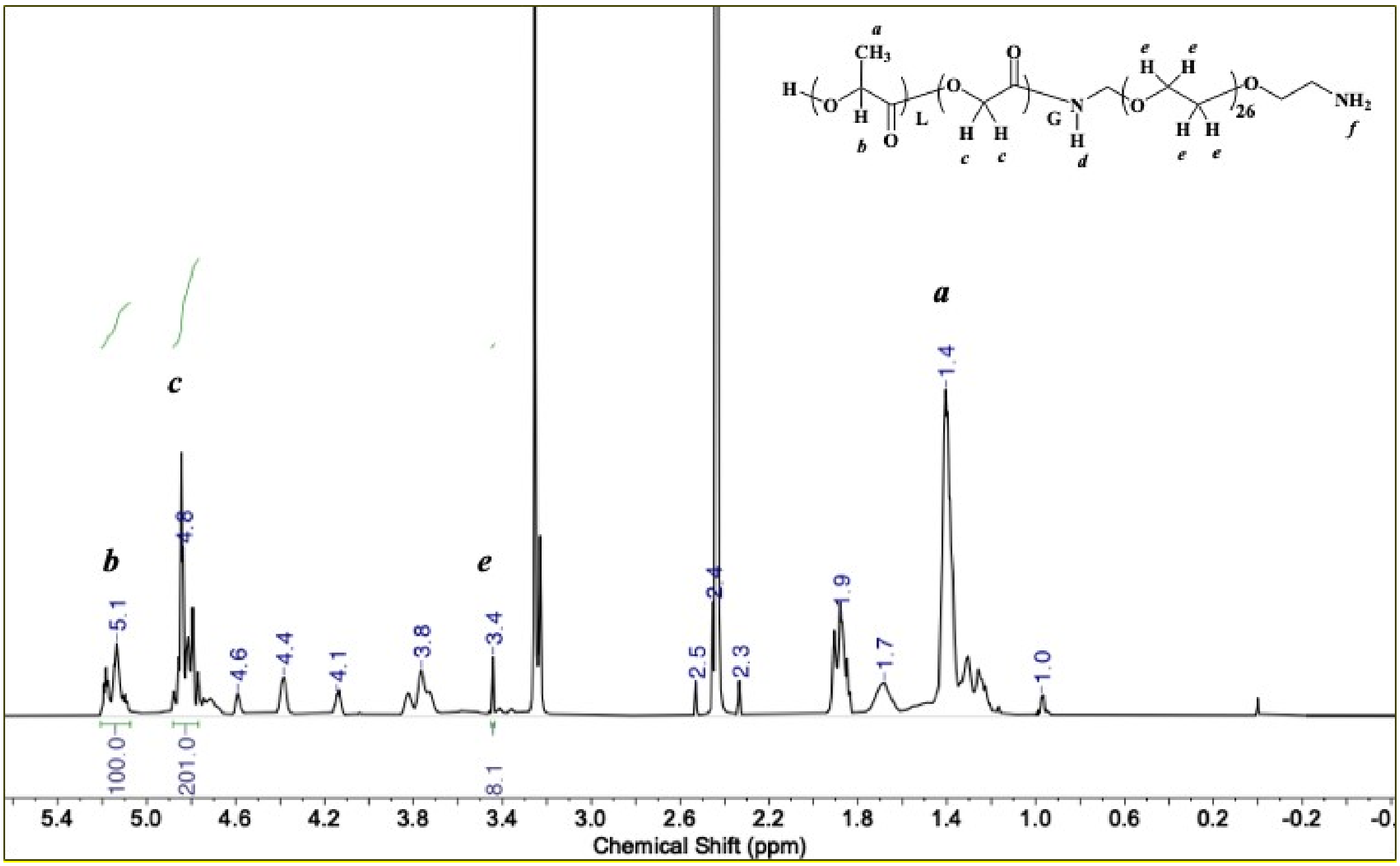

Figure S 7: ^{1}H NMR spectra of synthesized PEG26-PLGA NPs (700 MHz, d6-DMSO).

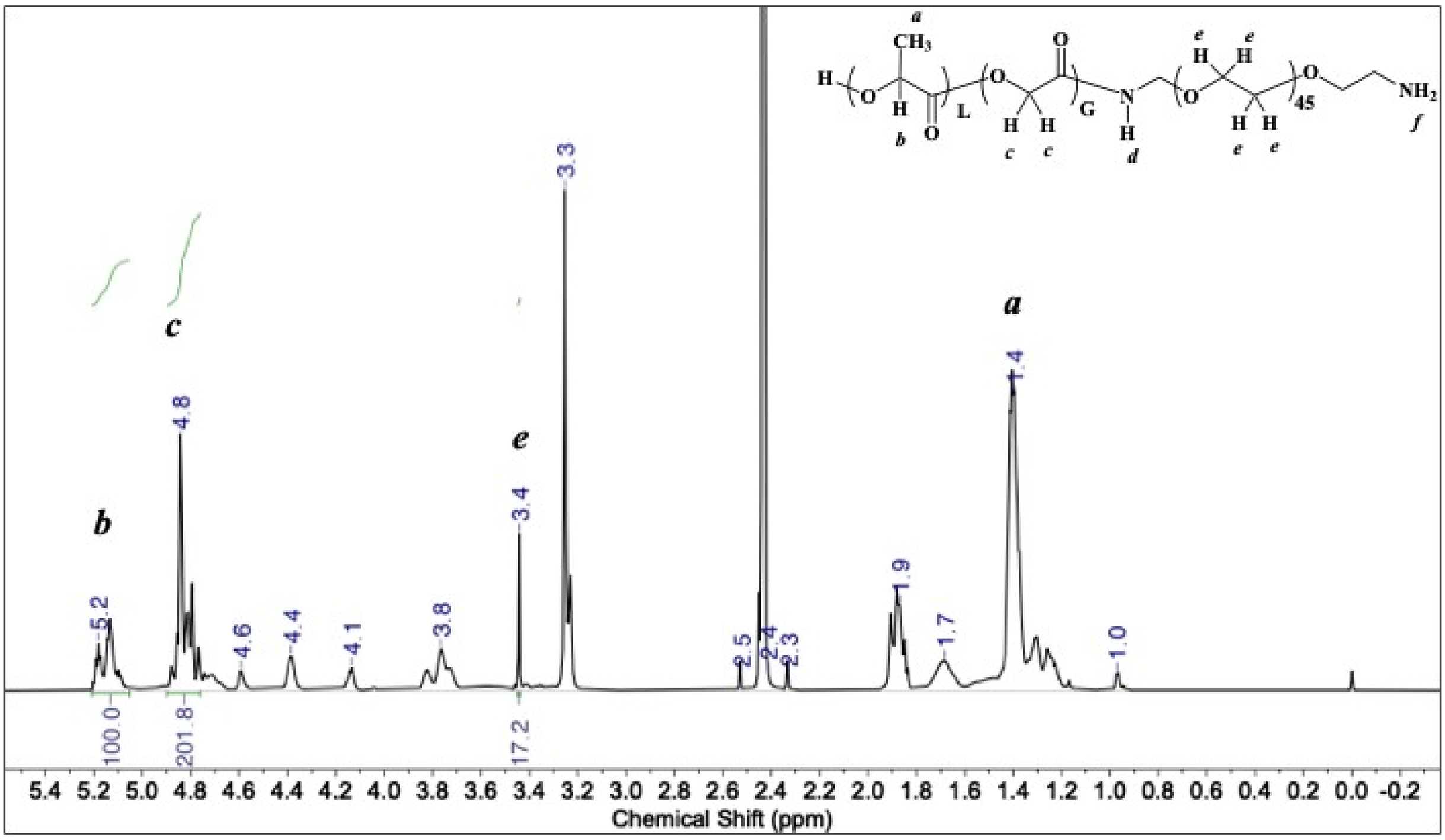

Figure S 8: ^{1}H NMR spectra of synthesized PEG45-PLGA NPs (700 MHz, d6-DMSO).

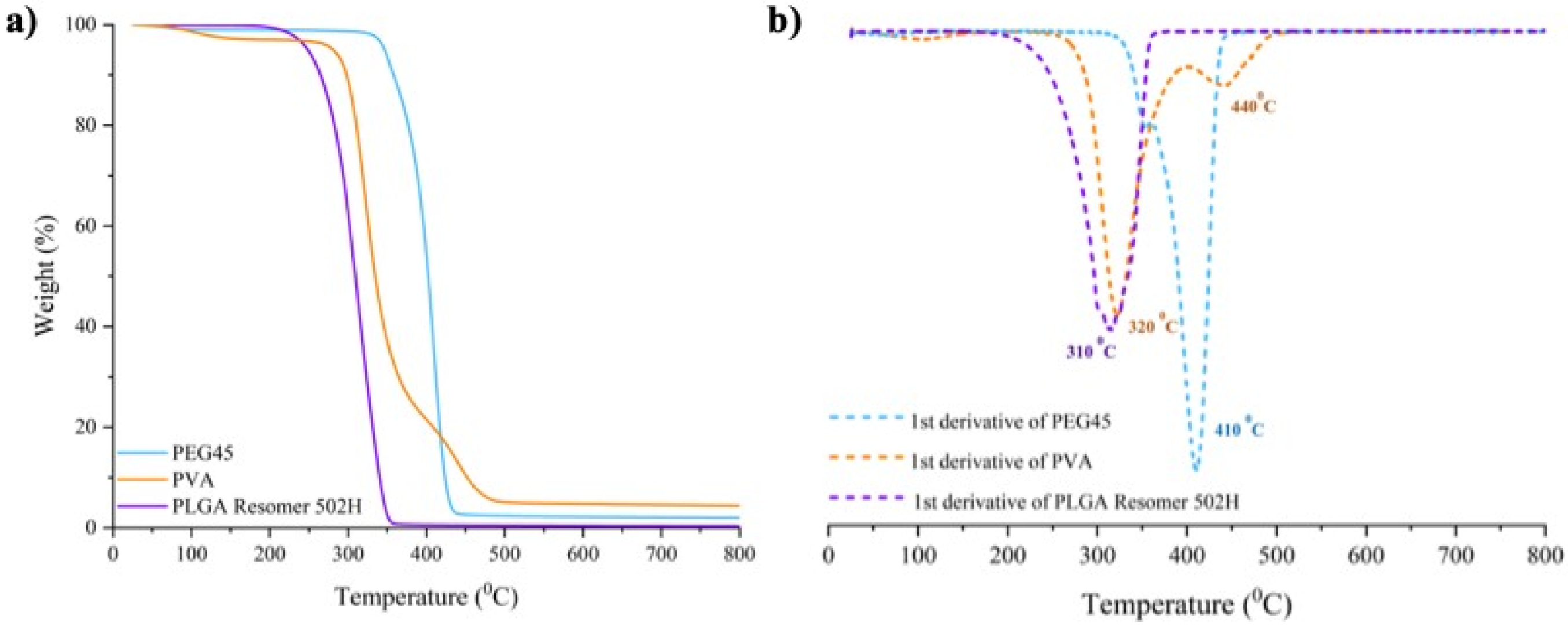

Figure S 9: a) TGA and **b)** DTG curves of PEG45, PVA, PLGA Resomer, and PLGA NPs

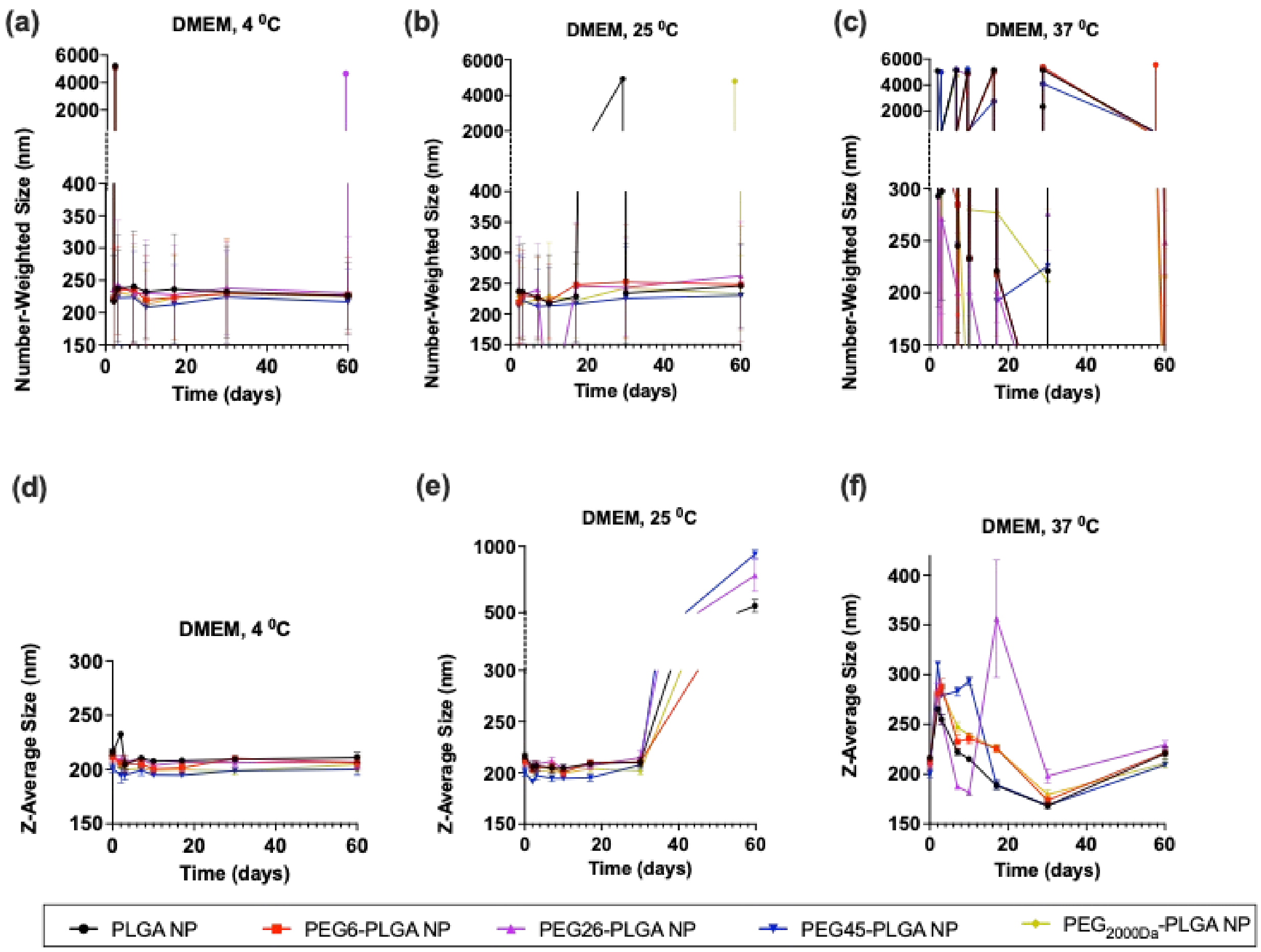

Figure S 10: Zoom-in stability results of PLGA NP (black), PEG6-PLGA NP (red), PEG26-PLGA NP (purple), PEG45-PLGA NP (blue), and PEG2000Da-PLGA NP (yellow) over 60 days in DMEM, presented as number-weighted particle size (nm) at **(a)** 4°C, **(b)** 25°C, and **(c)** 37°C, and Z-average mean size (nm) at **(d)** 4°C, **(e)** 25°C, and **(f)** 37°C.

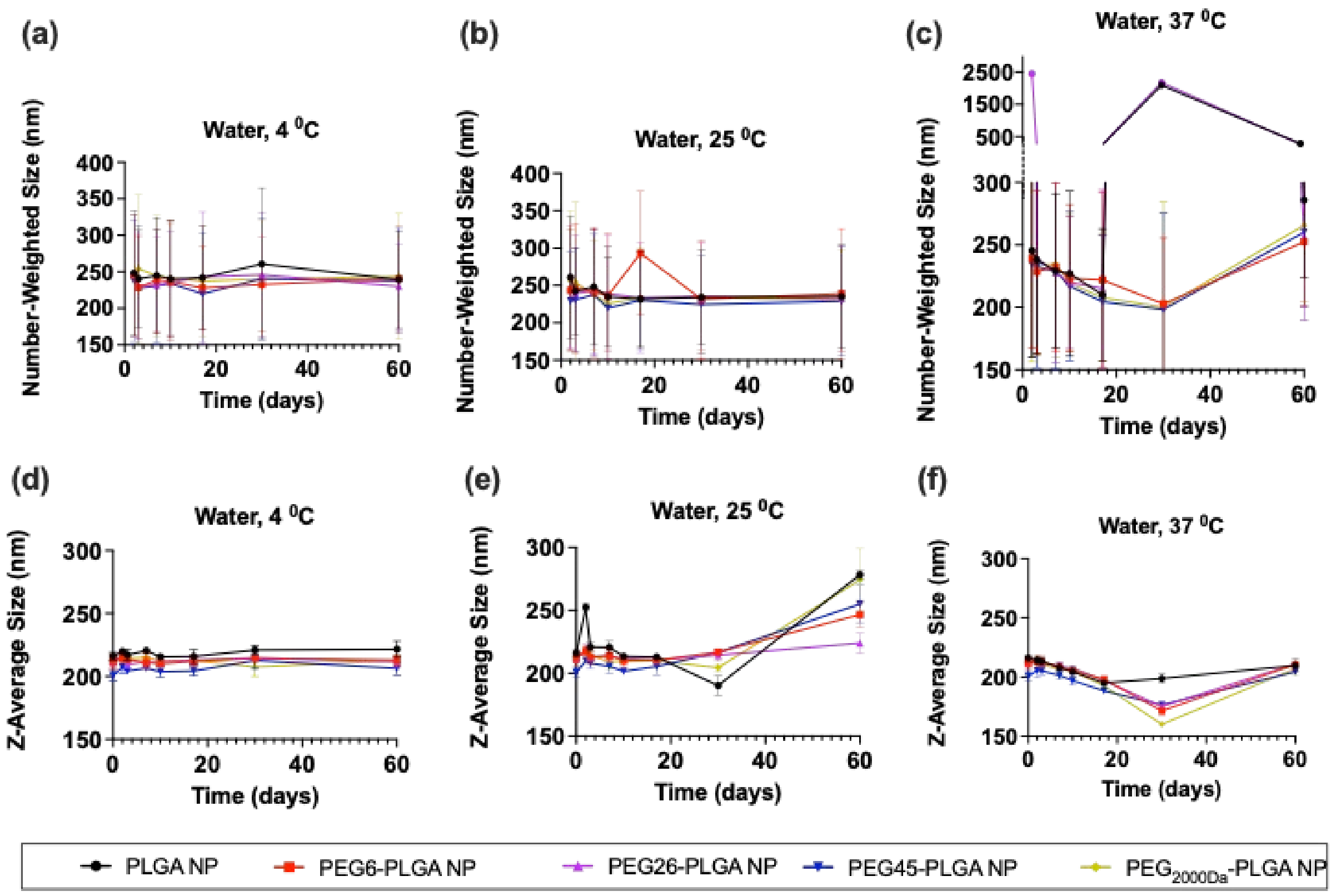

Figure S11: Zoom-in stability results of PLGA NP (black), PEG6-PLGA NP (red), PEG26-PLGA NP (purple), PEG45-PLGA NP (blue), and PEG_{2000Da}-PLGA NP (yellow) over 60 days in water, presented as number-weighted particle size (nm) at **(a)** 4°C, **(b)** 25°C, and **(c)** 37°C, and Z-average mean size (nm) at **(d)** 4°C, **(e)** 25°C, and **(f)** 37°C.

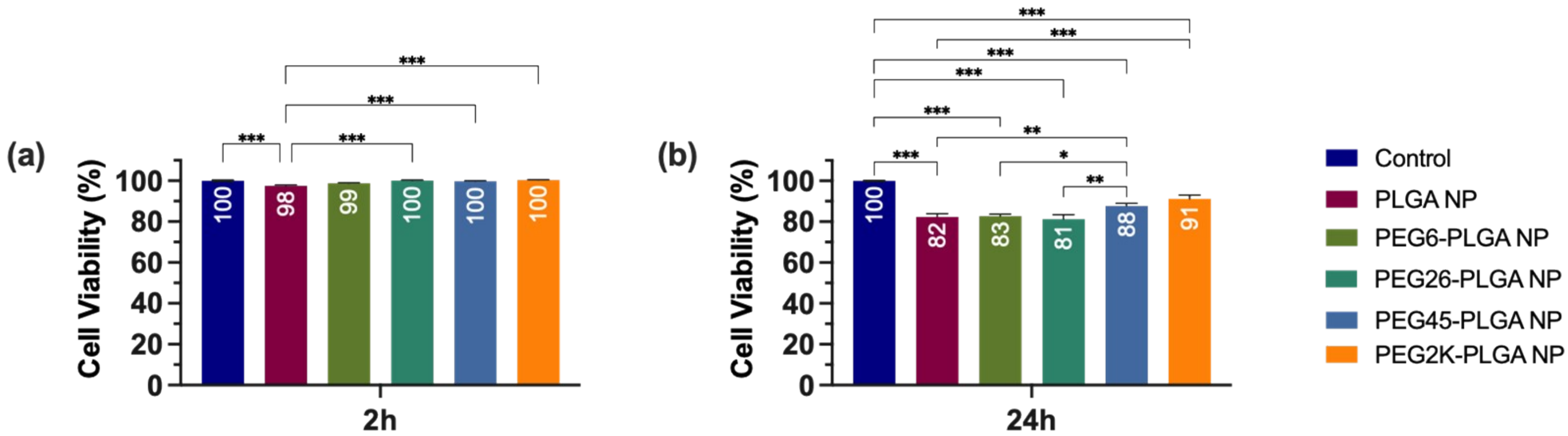

Figure S 12: Cell viability of PBMC after exposure to five different compositions for (a) 2h and (b) 24h. Data are presented as mean ± standard deviation (SD) from three independently prepared NP batches tested on cells from the same donor (n = 3), and normalized to the negative control as 100%.Statistical significance was determined by ordinary one-way ANOVA followed by Tukey's multiple-comparisons test; $p < 0.05$ (*), $p < 0.01$ (**), $p < 0.001$ (***).

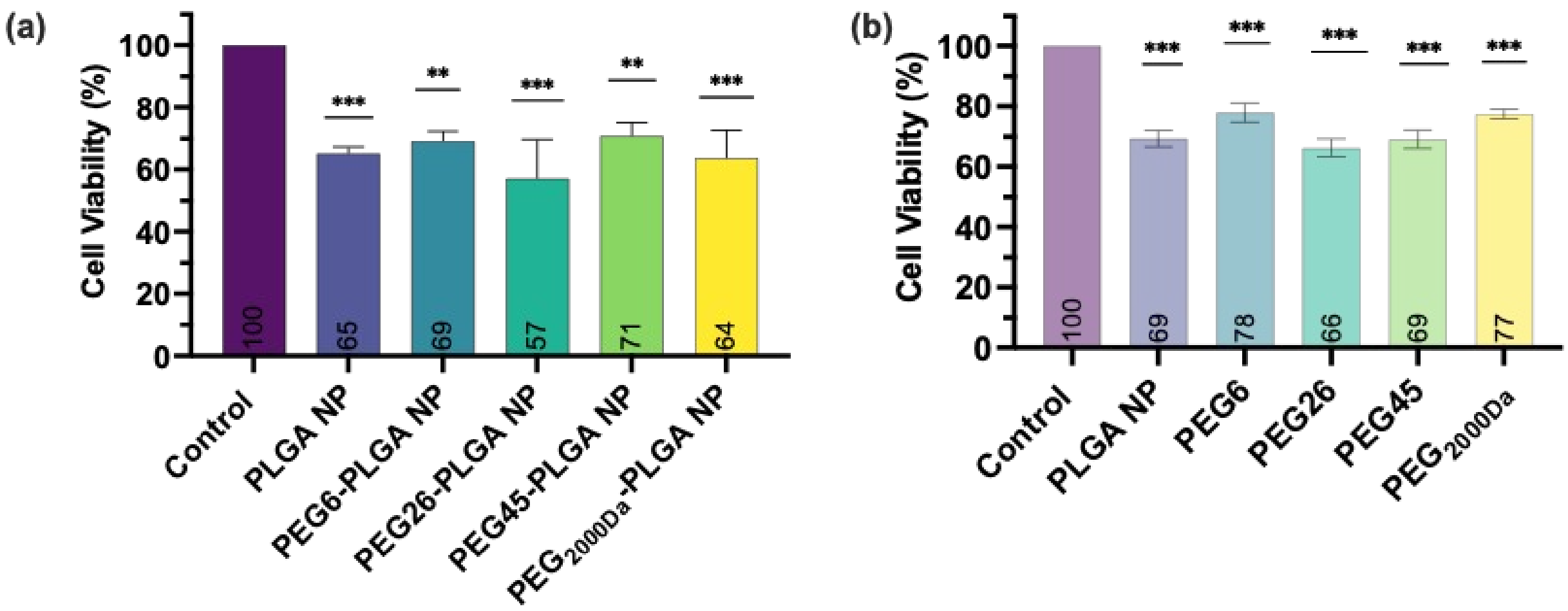

Figure S 13: Cell viability of RAW macrophages after exposure to different formulations for 4 hours: **(a)** PEG-PLGA nanoparticle components and **(b)** PEG itself. Data are presented as mean ± standard deviation (SD) from three independently prepared NP batches tested on RAW macrophages (n = 3) and normalized to the negative control as 100%. Statistical significance was determined by ordinary one-way ANOVA followed by Tukey's multiple-comparisons test; $p < 0.05$ (*), $p < 0.01$ (**), $p < 0.001$ (***).
